# Hepatoprotective Potential of Curcumin in the Prevention of Liver Dysfunction in a Porcine Model

**DOI:** 10.3390/nu18030408

**Published:** 2026-01-26

**Authors:** Kamila Kibitlewska, Varunkumar Asediya, Krzysztof Karpiesiuk, Urszula Czarnik, Marek Lecewicz, Paweł Wysocki, Prarthana Sharma, Iwona Otrocka-Domagała, Łukasz Zielonka, Andrzej Pomianowski, Adam Okorski, Garima Kalra, Sharmin Sultana, Nihal Purohit, Adam Lepczyński, Małgorzata Ożgo, Marta Marynowska, Agnieszka Herosimczyk, Elżbieta Redlarska, Brygida Ślaska, Krzysztof Kowal, Angelika Tkaczyk-Wlizło, Paweł Grychnik, Athul P. Kurian, Kaja Ziółkowska-Twarowska, Grzegorz Roman Juszczak, Mariusz Pierzchała, Katarzyna Chałaśkiewicz, Katarzyna Kępka-Borkowska, Ewa Poławska, Rafał Radosław Starzyński, Magdalena Ogłuszka, Hiroaki Taniguchi, Frieder Hadlich, Henry Reyer, Michael Oster, Nares Trakooljul, Avon Augustin Nalpadan, Siriluck Ponsuksili, Klaus Wimmers, Chandra Shekhar Pareek, Wojciech Kozera

**Affiliations:** 1 Department of Pig Breeding, Faculty of Animal Bioengineering, University of Warmia and Mazury in Olsztyn, 10-719 Olsztyn, Poland; kamila.kibitlewska@uwm.edu.pl (K.K.); krzysztof.karpiesiuk@uwm.edu.pl (K.K.); czar@uwm.edu.pl (U.C.); wojciech.kozera@uwm.edu.pl (W.K.); 2 Department of Infectious, Invasive Diseases and Veterinary Administration, Faculty of Biological and Veterinary Sciences, Institute of Veterinary Medicine, 87-100 Toruń, Poland; varun@doktorant.umk.pl (V.A.); garima-kalra@doktorant.umk.pl (G.K.); 503646@doktorant.umk.pl (S.S.); nihalpurohit13@doktorant.umk.pl (N.P.); 3 Centre for Modern Interdisciplinary Technologies, Nicolaus Copernicus University, 87-100 Toruń, Poland; 4 Department of Animal Biochemistry and Biotechnology, Faculty of Animal Bioengineering, University of Warmia and Mazury in Olsztyn, 10-719 Olsztyn, Poland; mlecew@uwm.edu.pl (M.L.); pawel.wysocki@uwm.edu.pl (P.W.); prarthana.sharma@student.uwm.edu.pl (P.S.); 5 Department of Pathological Anatomy, Faculty of Veterinary Medicine, University of Warmia and Mazury in Olsztyn, 10-719 Olsztyn, Poland; i.otrocka-domagala@uwm.edu.pl; 6 Department of Veterinary Prevention and Feed Hygiene, Faculty of Veterinary Medicine, University of Warmia and Mazury in Olsztyn, M. Oczapowskiego St. 13, 10-718 Olsztyn, Poland; lukasz.zielonka@uwm.edu.pl; 7 Internal Medicine Department, Faculty of Veterinary Medicine, University of Warmia and Mazury in Olsztyn, 10-719 Olsztyn, Poland; andrzej.pomianowski@uwm.edu.pl; 8 Department of Entomology, Phytopathology and Molecular Diagnostics, Faculty of Agriculture and Forestry, University of Warmia and Mazury in Olsztyn, Plac Łódzki 5, 10-727 Olsztyn, Poland; adam.okorski@uwm.edu.pl; 9 Department of Physiology, Cytobiology and Proteomics, West Pomeranian University of Technology in Szczecin, 70-310 Szczecin, Poland; adam.lepczynski@zut.edu.pl (A.L.); malgorzata.ozgo@zut.edu.pl (M.O.); marta.marynowska@zut.edu.pl (M.M.); agnieszka.herosimczyk@zut.edu.pl (A.H.); elzbieta_lichwiarska@zut.edu.pl (E.R.); 10 Institute of Biological Bases of Animal Production, Faculty of Animal Sciences and Bioeconomy, University of Life Sciences in Lublin, 20-950 Lublin, Poland; brygida.slaska@up.lublin.pl (B.Ś.); krzysztof.kowal@up.lublin.pl (K.K.); angelika.tkaczyk@up.lublin.pl (A.T.-W.); pawel.grychnik@up.lublin.pl (P.G.); athul.kurian@up.lublin.pl (A.P.K.); kaja.ziolkowska@up.lublin.pl (K.Z.-T.); 11 Department of Animal Behavior and Welfare, Institute of Genetics and Animal Biotechnology of the Polish Academy of Sciences, 05-552 Jastrzębiec, Poland; g.juszczak@igbzpan.pl; 12 Department of Genomics and Biodiversity, Institute of Genetics and Animal Biotechnology of the Polish Academy of Sciences, 05-552 Jastrzębiec, Poland; m.pierzchala@igbzpan.pl (M.P.); k.chalaskiewicz@igbzpan.pl (K.C.); k.kepka@igbzpan.pl (K.K.-B.); e.polawska@igbzpan.pl (E.P.); m.ogluszka@igbzpan.pl (M.O.); 13 Department of Molecular Biology, Institute of Genetics and Animal Biotechnology of the Polish Academy of Sciences, 05-552 Jastrzębiec, Poland; r.starzynski@igbzpan.pl; 14 Department of Experimental Embryology, Institute of Genetics and Animal Biotechnology of the Polish Academy of Sciences, 05-552 Jastrzębiec, Poland; h.taniguchi@igbzpan.pl; 15 African Genome Center, Mohammed VI Polytechnic University, UM6P, Lot 660, Hay Moulay Rachid, Ben Guerir 43150, Morocco; 16 Research Institute for Farm Animal Biology (FBN), Wilhelm-Stahl-Allee 2, 18196 Dummerstorf, Germany; hadlich@fbn-dummerstorf.de (F.H.); oster@fbn-dummerstorf.de (M.O.); trakooljul@fbn-dummerstorf.de (N.T.); nalpadan@fbn-dummerstorf.de (A.A.N.); s.wimmers@fbn-dummerstorf.de (S.P.); wimmers@fbn-dummerstorf.de (K.W.); 17 Faculty of Agricultural, Civil and Environmental Engineering, University of Rostock, 18059 Rostock, Germany

**Keywords:** curcumin, hepatitis, liver, pig, liver dysfunction, oxidative stress, hepatoprotective effect, hepatocyte

## Abstract

Curcumin, the major polyphenolic constituent of *Curcuma longa*, has been widely investigated as a hepatoprotective adjunct due to its antioxidant and immunomodulatory properties. This review evaluates the relevance of curcumin for the prevention and management of liver dysfunction and hepatitis in pigs by synthesizing available porcine evidence and integrating mechanistic insights from translational liver injury models where pig-specific data remain limited. Across experimental hepatic injury contexts, curcumin administration is most consistently associated with reduced biochemical and structural indicators of hepatocellular damage, including decreased aminotransferase activity, attenuation of lipid peroxidation, and enhancement of endogenous antioxidant defenses. These effects are mechanistically linked to suppression of pro-inflammatory signaling pathways, particularly NF-κB-related transcriptional activity and inflammasome-associated responses, together with reduced expression of key cytokines such as TNF-α, IL-1β, and IL-6. Concurrent activation of Nrf2-centered cytoprotective pathways and induction of phase II antioxidant enzymes (including HO-1, GST, and NQO1) appear to constitute a conserved axis supporting hepatic oxidative stress resilience. In swine-relevant infectious settings, available data further support antiviral activity against selected porcine pathogens, including classical swine fever virus and porcine reproductive and respiratory syndrome virus, potentially mediated through interference with lipid-dependent stages of viral replication and modulation of Kupffer cell activation. Although combination strategies with established hepatoprotective approaches are conceptually attractive, current synergy evidence remains heterogeneous and largely extrapolated. Overall, curcumin represents a plausible adjunct candidate for supporting porcine liver health; however, translation into practice will depend on resolving formulation-dependent bioavailability constraints and strengthening the pig-specific evidence base.

## 1. Introduction

The liver is a multifunctional abdominal organ whose physiology depends on coordinated interactions between parenchymal and non-parenchymal cell populations. Hepatocytes constitute the dominant parenchymal compartment (approximately 70–80% of liver cells and ~90% of liver mass) and execute the core tasks of bile production and integrated carbohydrate, lipid, and protein metabolism. Despite retaining proliferative capacity, hepatocytes exhibit very low baseline turnover; under physiological conditions, most remain in the quiescent G0 phase, and only a minor fraction enters DNA synthesis. Cholangiocytes, although epithelial and derived from hepatoblasts, are not classified as parenchymal cells; they line the biliary ducts, represent approximately 3–5% of hepatic cells, and contribute to bile formation through water and electrolyte secretion, accounting for a substantial share of bile volume. The non-parenchymal compartment comprises mesoderm-derived cells that shape hepatic immunity, microcirculation, and tissue remodeling, including Kupffer cells (resident macrophages responsible for erythrophagocytosis, immune surveillance, and inflammatory regulation), hepatic stellate cells (central regulators of extracellular matrix turnover, fibrosis, and regeneration), liver sinusoidal endothelial cells (specialized endothelium controlling sinusoidal permeability, endocytosis, and paracrine signaling), and pit cells (liver-resident natural killer cells contributing to innate immunity), supported by stromal elements such as fibroblasts and smooth muscle cells. Structurally, the hepatic lobule is the basic anatomical and functional unit, organized around a central vein and divided into three metabolic zones (zones 1–3) based on proximity to the portal triad and central vein [1].

**Figure 1:**
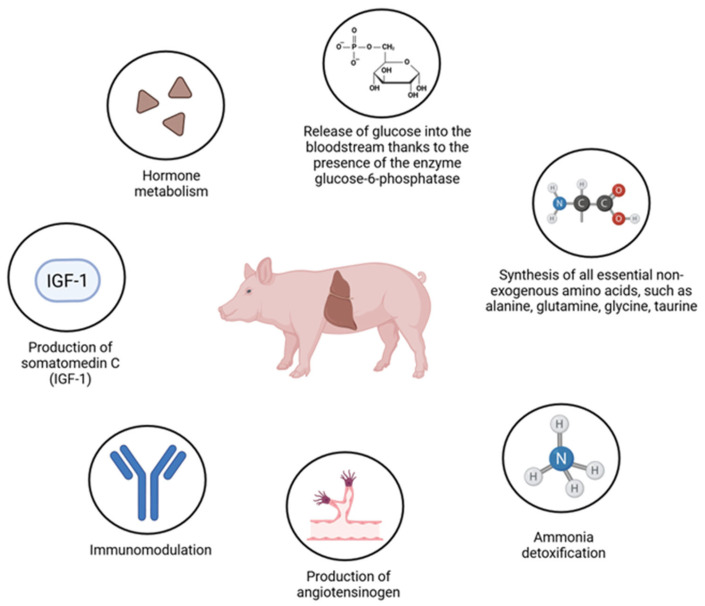
Key physiological functions of the porcine liver. The liver in pigs performs a wide spectrum of essential metabolic, endocrine and immunological functions. The figure has been created with www.biorender.com (accessed on 1 May 2025).

Across animal species, the liver functions as the principal metabolic hub that buffers nutrient availability and maintains systemic homeostasis. The major metabolic, endocrine, and immunological functions of the porcine liver are summarized in Figure 1. In carbohydrate metabolism, the liver stabilizes blood glucose through postprandial glycogen synthesis and, during fasting, through glycogenolysis and gluconeogenesis using amino acids, glycerol, and lactate [2]. In lipid metabolism, it synthesizes cholesterol and fatty acids, assembles lipoproteins for lipid transport, and converts excess cholesterol into bile acids secreted into the intestine; importantly, the relative contribution of hepatic lipogenesis varies by species, being predominant in birds and shared with adipose tissue in mammals [3]. In protein metabolism, hepatic deamination generates ammonia, which mammals detoxify through the urea cycle (ornithine cycle), whereas birds and many reptiles generate uric acid; the liver also sustains systemic protein homeostasis by synthesizing endogenous amino acids and producing major plasma proteins, including albumin, fibrinogen, prothrombin, and other coagulation factors [4].

A wide range of plant-derived compounds, including silymarin from *Silybum marianum*, glycyrrhizin from *Glycyrrhiza glabra*, polyphenols from green tea, and various fermented phytoproducts, have been reported to exert hepatoprotective effects through antioxidant, anti-inflammatory, and metabolic regulatory mechanisms. Rather than providing a comprehensive comparison of all hepatoprotective phytochemicals, the present review focuses on curcumin as a representative compound with unusually broad mechanistic coverage across redox regulation, inflammatory signaling, immune modulation, and metabolic pathways. This focus is further justified by the depth of mechanistic characterization available for curcumin and the presence of emerging porcine-specific in vivo evidence, particularly in oxidative stress—and endotoxin-associated hepatic injury models. Other phytochemicals clearly warrant continued investigation; however, curcumin provides a uniquely well-characterized framework for discussing translational challenges and opportunities in porcine liver health.

Although porcine hepatic architecture and many functions are broadly comparable to those of other mammals, pigs display distinctive metabolic characteristics that are directly relevant to both disease biology and translational interpretation. In carbohydrate handling, glycogenesis and glycogenolysis proceed similarly to other mammals, but the porcine liver contributes relatively little to postprandial glucose clearance, with a greater share of glucose uptake occurring in peripheral tissues such as skeletal muscle and adipose tissue. Moreover, hepatic conversion of glucose to fatty acids is minimal in pigs, reflecting limited processing of glucose into fatty acids by porcine hepatocytes compared with species such as rodents and humans. Nevertheless, pigs retain the canonical hepatic capacity to export glucose into circulation via glucose-6-phosphatase, which converts glucose-6-phosphate to free glucose [5]. In lipid metabolism, pigs exhibit limited hepatic de novo synthesis of long-chain fatty acids because lipogenesis occurs predominantly in adipose tissue and is associated with low hepatic acetyl-CoA carboxylase activity [6]. A further distinguishing feature is minimal ketogenesis due to the absence of mitochondrial 3-hydroxy-3-methylglutaryl-coenzyme A synthase; consequently, ketone bodies such as acetone, acetoacetyl-CoA, and β-hydroxybutyrate are not produced to the extent observed in many other mammals (including humans, ruminants, and rodents), in which ketone concentrations can rise markedly during starvation or diabetes [7]. In addition, the porcine liver synthesizes several biologically essential non-essential amino acids (including alanine, glutamine, glycine, and taurine) using other amino acids as substrates, with methionine serving as a Sulphur donor for cysteine and taurine synthesis [8].

Porcine hepatic xenobiotic metabolism is broadly similar to that of other mammals. The cytochrome P450 enzyme profile in pigs is reported to resemble that of humans, which supports the widespread use of pigs as translational models for drug metabolism [9]. However, species-specific vulnerabilities remain practically important; for example, pigs may show insufficiently effective metabolism and elimination of aflatoxins, increasing susceptibility to aflatoxicosis and making feed contamination a relevant hepatic risk factor in pig production systems [10]. Beyond metabolism, the liver also functions as an endocrine organ. Hepatocytes produce somatomedin C (IGF-1) in response to growth hormone stimulation and synthesize angiotensinogen, a liver-derived prohormone converted in circulation to angiotensin I and subsequently angiotensin II, thereby contributing to blood pressure regulation and sodium metabolism [11]. The liver is also characterized by high regenerative capacity; while this is well documented in mammals such as rats and humans after partial hepatectomy, pigs similarly demonstrate rapid post-resection growth and are considered valuable experimental models for studying liver regeneration [12].

Within this physiological and metabolic context, hepatitis in pigs is defined as an inflammatory process occurring within the liver parenchyma that can arise from diverse aetiologies, including viral, bacterial or parasitic infections, toxin exposure, and metabolic or dietary disturbances. Inflammatory injury can compromise key hepatic functions and may present with non-specific clinical signs such as apathy, reduced appetite, weight loss, fever, bruising, abdominal or limb swelling and elevated liver enzyme activity. Among hepatotropic viruses of major relevance in pigs are the hepatitis E virus (HEV) and porcine circovirus type 2 (PCV2). HEV genotypes 3 and 4 typically cause asymptomatic infections that are controlled by host immunity and transmitted primarily via the fecal–oral route. Lymphocyte-mediated targeting of infected hepatocytes is implicated in inflammatory changes, and serological dynamics generally include early IgM responses followed by IgG responses during recovery, consistent with activation of humoral immunity [13]. Histopathological descriptions include multifocal perilobular inflammation with lymphoplasmacytic infiltration and occasional foci of hepatocyte necrosis, often without obvious macroscopic lesions [14]. Concordantly, biochemical parameters usually remain within reference ranges or show only minor changes; experimental infections generally do not induce marked elevations of ALT or AST, although slight transient ALT increases have been observed during viremia in a subset of animals [15]. Importantly, asymptomatic carriages have substantial epidemiological relevance.

Curcumin, as a bioactive dietary polyphenol, has been widely investigated for its hepatoprotective and immunomodulatory properties; however, evidence relevant to porcine liver dysfunction remains scattered and uneven in scope. While pigs represent an important translational model for hepatic pathology and a species of direct agricultural relevance, available porcine studies are limited in number and methodological consistency and are often interpreted in isolation from mechanistic insights derived from other liver-injury models.

The aim of this narrative review was to critically synthesize and contextualize existing evidence on the effects of curcumin in porcine liver dysfunction and hepatitis. To address gaps in pig-specific data, findings from translational experimental liver-injury models were integrated to elucidate conserved molecular pathways underlying curcumin’s antioxidant, anti-inflammatory, and cytoprotective actions. By systematizing biochemical, histopathological and immunological outcomes across diverse hepatic injury contexts, this review seeks to clarify the relevance of curcumin as a hepatoprotective adjunct in swine, identify key limitations related to bioavailability and study design, and highlight priorities for future controlled porcine intervention studies.

## 2. Methods

This review was conducted as a structured narrative synthesis to evaluate the biological activity, pharmacokinetics, and hepatoprotective potential of curcumin (*Curcuma longa* L.) and defined curcuminoid preparations. The primary focus was hepatic injury and dysfunction, including biochemical, histopathological, oxidative stress, inflammatory, and fibrosis-related endpoints. Because the overarching scope of the manuscript concerns porcine liver dysfunction and hepatitis, porcine studies and swine-relevant contexts were prioritized where available; evidence from other mammalian liver models was incorporated to support mechanistic interpretation and translational plausibility when porcine data were limited.

### 2.1. Search Strategy and Information Sources

Relevant literature was identified through searches of MEDLINE (PubMed), Embase, and Web of Science from database inception to November 2025. Search queries combined controlled vocabulary (where applicable) and free-text terms and were constructed using Boolean operators. Keywords included combinations of: “curcumin,” “*Curcuma longa*,” “turmeric,” “curcuminoids,” “polyphenols,” “hepatoprotection,” “liver injury,” “liver disease,” and “pharmacokinetics.” To enhance completeness, the reference lists of eligible articles and relevant recent reviews were screened, and additional studies were identified through citation tracking of key records where needed.

### 2.2. Eligibility Criteria

Studies were included if they met all of the following criteria:

(i) Original, peer-reviewed in vivo or in vitro research;
(ii) Evaluation of curcumin or a defined curcuminoid preparation as the primary intervention;
(iii) Sufficient characterization of the tested compound to permit interpretation, including explicit identification of source and, where available, purity and composition and/or formulation details (e.g., standardized extract, nanoparticle formulation, phospholipid complex, or other delivery system);
(iv) Reporting of at least one quantitative hepatic endpoint, such as circulating liver enzymes (alanine aminotransferase (ALT), aspartate aminotransferase (AST), alkaline phosphatase (ALP), gamma-glutamyl transferase (GGT)), bilirubin, oxidative stress indices, inflammatory mediators/cytokines, and/or histological outcomes (e.g., steatosis, necrosis, fibrosis);
(v) Adequate methodological detail to interpret dose, route, timing/duration, model context, and the principal outcomes.

Studies were excluded if they:

(i) Lacked essential methodological details (e.g., undefined dosing regimen, route, or injury model);
(ii) Did not specify curcumin/curcuminoid composition, purity, or formulation sufficiently to enable interpretation;
(iii) Were non-original works (editorials, letters, commentaries);
(iv) Did not report hepatic outcomes or did not provide quantifiable liver-related endpoints.

### 2.3. Data Extraction and Outcome Domains

From each eligible study, data were extracted on:

(i) Experimental model characteristics (e.g., toxin-induced injury, diet-induced steatosis, ischemia–reperfusion, inflammatory and/or metabolic models; and, where relevant, swine pathogens or swine-relevant contexts);
(ii) Intervention parameters (compound identity, formulation/delivery system, dose, timing, duration, and route of administration);
(iii) Outcomes within predefined domains: biochemical indicators of liver injury and function, histopathology (including fibrosis/steatosis/necrosis when reported), oxidative stress measures, inflammatory signaling readouts, and molecular markers related to fibrosis, regeneration, or hepatocyte survival.

### 2.4. Synthesis Approach and Evidence Weighting

Given heterogeneity across species, models, curcumin formulations, dosing strategies, and endpoint definitions, meta-analysis was not performed. Instead, findings were synthesized qualitatively and structured by model category and outcome domain. Interpretation explicitly considered internal validity, exposure plausibility, and translational relevance. Greater weight was assigned to studies meeting the following criteria:

(i) Use of standardized or well-characterized curcumin preparations with defined composition and formulation;
(ii) Clear, reproducible, and clinically interpretable hepatic endpoints (biochemistry and/or histology);
(iii) Reporting that enabled linkage between delivery and effect (e.g., pharmacokinetic data, tissue distribution, or formulation-specific bioavailability);
(iv) Porcine in vivo evidence or studies directly aligned with porcine liver dysfunction/hepatitis.

Throughout the review, mechanistic conclusions are explicitly categorized as: (a) directly supported by porcine liver tissue/cell evidence, or (b) inferred from non-porcine liver models based on conserved signaling. Inferred mechanisms are stated as such and not presented as experimentally validated in pigs.

Methodological limitations—including incomplete reporting, unclear dosing, inadequate controls, and absence of standardized histological assessment—were considered when judging the strength and generalizability of evidence. Where results were inconsistent, discrepancies were highlighted and interpreted in the context of model differences, formulation/dose, and endpoint selection. Conclusions were framed according to an explicit evidence hierarchy, prioritizing porcine in vivo studies, followed by porcine ex vivo/in vitro data, then non-porcine liver models, and finally non-hepatic mechanistic studies.

### 2.5. Study Selection and Control of Selection Bias

To minimize selection bias and enhance transparency, study identification and selection were conducted using predefined criteria applied uniformly across all records. Titles and abstracts retrieved from database searches were screened independently by two authors to assess potential eligibility. Full texts of potentially relevant articles were then evaluated against the inclusion and exclusion criteria specified in Section 2.2. Discrepancies at any stage of screening were resolved through discussion and consensus, with reference to the predefined eligibility framework to ensure consistent application of selection rules.

The review followed structured, PRISMA-informed principles appropriate for narrative syntheses, including systematic database searching, explicit eligibility criteria defined a priori, and transparent documentation of reasons for exclusion. Inclusion and exclusion decisions were based on methodological and reporting considerations rather than study outcomes, thereby reducing the risk of outcome-driven selection. This approach was intended to ensure balanced representation of available evidence while maintaining alignment with the review’s scope and translational focus on porcine liver dysfunction and hepatoprotection.

To enhance transparency of the narrative synthesis, studies were qualitatively appraised using predefined indicators of internal validity, robustness, and translational relevance, which guided evidence weighting as summarized in Table 1.

## 3. Mechanism of Liver Dysfunctions in Pigs

**Figure 2:**
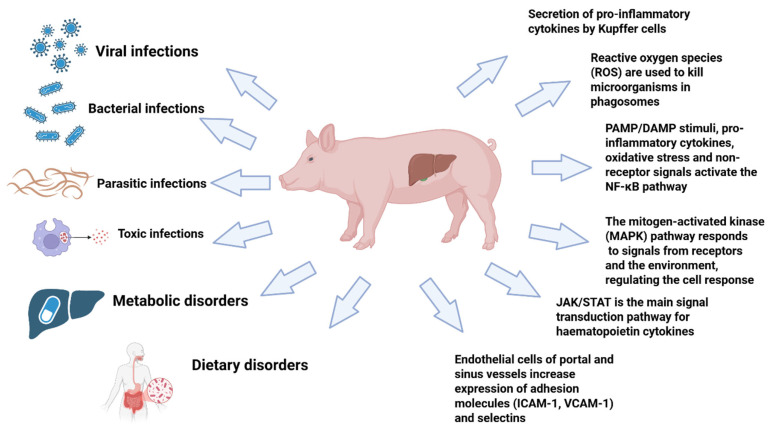
Etiology and molecular mechanisms of liver dysfunctions in swine. Hepatitis in pigs arises from diverse etiological factors, including viral, bacterial and parasitic infections, exposure to toxins, as well as metabolic and dietary disorders. Together, these mechanisms underpin the development and progression of hepatitis in swine, regardless of the initial cause. Figure has been created with www.biorender.com (accessed on 1 May 2025).

Porcine hepatitis and liver dysfunction are initiated by diverse aetiologies—viral infection, systemic bacterial dissemination, and hepatotoxic feed contaminants—but they largely converge on the same intrahepatic pathophysiological axis: Kupffer-cell activation and cytokine amplification, oxidative stress, hepatocyte death programs, and tissue remodeling with fibrogenic progression if injury persists. This convergence is critical for intervention logic because it identifies tractable nodes (NF-κB/MAPK/JAK/STAT signaling and redox imbalance) that sit upstream of hepatocellular injury and fibrosis. Accordingly, the major etiological categories and shared molecular mechanisms implicated in porcine liver dysfunction are summarized in Figure 2. Additionally, A comparative overview of these major infectious and toxic triggers and their shared mechanistic outputs is provided in Table 2.

### 3.1. Etiological Landscape: Representative Triggers of Porcine Hepatic Inflammation

In infection-related liver injury models, it is important to distinguish between direct antiviral effects of curcumin, which require demonstration of altered viral replication or burden, and indirect hepatoprotective effects mediated by immunomodulation, reduced macrophage activation, and attenuation of inflammatory signaling.

HEV (zoonosis and subclinical hepatic inflammation): Pigs are a reservoir of zoonotic HEV genotypes 3 and 4 and may infect humans, most often via consumption of undercooked pork (particularly pig liver) or direct contact with infected pigs [13]. Contamination of retail liver and muscle products has been documented; in Japan, HEV was detected in approximately 2% of tested livers intended for consumption [14].
PCV2 (systemic infection with hepatic apoptosis and fibrogenic potential): PCV2, a key driver of Postweaning Multisystemic Wasting Syndrome/Porcine Circovirus–Associated Disease (PMWS/PCVAD), replicates primarily in immune cells but also in hepatocytes, making the liver a frequent target organ. PCV2 DNA has been detected in a large proportion of liver samples from PMWS-affected pigs, with macrophages and hepatocytes identified as infected populations [16]. Intrahepatic viral load correlates with hepatocyte apoptosis, supported by co-localization of viral signal and active caspase-3 [17]. Histopathology includes lobular inflammation with lymphocytic–histiocytic infiltration, scattered apoptotic necrosis, architectural disorganization, and—when sustained—periportal/perilobular fibrosis [18]. Liver enzymes are not consistently used diagnostically in PCV2-associated hepatitis [16], but severe cases may present with parenchymal jaundice linked to extensive injury and intrahepatic cholestasis [19].
Bacterial dissemination (septicemic hepatoinflammation): In pigs, hepatic inflammation secondary to bacterial infection commonly reflects systemic spread. *Salmonella enterica* typically invades Peyer’s patches, disseminates via mesenteric lymph nodes, and reaches the bloodstream [20]. Because it survives within macrophages, septicaemia and generalized disease are common, with liver involvement prominent in pigs [19]. Hepatic lesions include scattered necrotic foci (paratyphoid nodules) representing microabscess/granuloma formation dominated by macrophages and neutrophils; chronic granulomas are described but acute/hyperacute disease is more typical [20]. While aminotransferases may rise moderately, systemic effects predominate and residual chronic hepatic dysfunction after recovery is generally limited, whereas impacts on performance and food safety are economically important [19].
Leptospirosis (necrosis–cholestasis pattern with systemic consequences): *Leptospira* spp. cause a zoonotic systemic infection in pigs (commonly Pomona and Bratislava), acquired from urine- or water-contaminated environments with subsequent leptospiremia and dissemination. The liver is a major target organ [21]. Injury reflects combined endothelial and hepatocellular damage through toxic-enzymatic and immune-mediated mechanisms, with necrosis and cholestatic changes and corresponding ALT/ALP elevations. Disease importance is driven by high neonatal mortality and reproductive losses in sows [21].
Mycotoxins (aflatoxin as the canonical hepatotoxic exposure): In farm conditions, toxic hepatitis frequently reflects feed-borne mycotoxins, with aflatoxins among the most potent. After ingestion, aflatoxin B_1_ (AFB_1_) is transported to the liver and bioactivated by cytochrome P450 enzymes to a reactive epoxide that binds DNA and proteins, producing cytotoxic, genotoxic, and immunosuppressive effects. In pigs, major consequences include reduced weight gain, impaired feed utilization, reproductive disturbances, and increased susceptibility to infections, even at subclinical exposures [22]. Acute injury preferentially affects centrilobular regions, and liver enzyme activity rises rapidly; Miller et al. reported increases in ICD, ALP, SDH, and AST within 24 h in most exposed piglets [23]. Chronic exposure can progress to cirrhosis and hepatocellular carcinoma, although such outcomes are rarely recognized in commercial systems due to slaughter age [22].

### 3.2. Cellular Control Layer: Kupffer Cells Couple Exposure Sensing to Inflammation and Fibrosis

Kupffer cells (KC) function as the principal intrahepatic sensor–effector population for blood-borne pathogens, microbial products, and toxins. Upon activation, KC rapidly secrete TNF-α, IL-1, and IL-6 and thereby initiate and amplify local inflammation [23]. TNF-α and IL-1 drive endothelial activation, vascular permeability, and leukocyte recruitment; IL-6 is a key regulator of the hepatic acute-phase response by inducing synthesis of proteins such as CRP, serum amyloid A, and haptoglobin while suppressing albumin production [24]. KC also limit secondary injury by clearing apoptotic debris and processing antigens [25]. At the same time, KC provides a mechanistic bridge from inflammation to remodeling by recruiting and activating hepatic stellate cells (HSC), including through TGF-β1–linked signaling [26].

### 3.3. Hepatocyte Death Programs as an Inflammatory “Switch”

Hepatocyte death during hepatitis occurs primarily through apoptosis and necrosis (and, under defined conditions, pyroptosis). Apoptosis is regulated and limits extracellular spillover, whereas necrosis involves membrane rupture and release of DAMPs (e.g., HMGB1, ATP, mitochondrial DNA), which strongly activate KC and infiltrating leukocytes and can generate feed-forward inflammatory loops [27]. In severe toxic injury and acute hepatitis, these amplification dynamics can contribute to extensive parenchymal damage and escalating cytokine signaling [28,29]. In chronic states, inflammatory infiltrates often localize to portal regions, where cytokines such as TNF-α and IL-1β induce adhesion pathways (ICAM-1, VCAM-1, selectins) that support leukocyte rolling, adhesion, and transmigration [23]. Dendritic cells coordinate adaptive immunity via antigen presentation and cytokine-driven polarization, while plasmacytoid dendritic cells can establish antiviral states via type I interferons [30]. T lymphocytes dominate hepatic infiltrates, with CD8^+^ cytotoxic cells eliminating infected hepatocytes via perforin–granzyme or Fas–FasL pathways; NK/NKT populations contribute cytotoxicity and cytokine signaling, including IFN-γ and TNF. Ectopic lymphoid structures may occur in prolonged inflammation, though less frequently in pigs than in humans [31].

### 3.4. Remodeling and Fibrosis: Portal Fibroblasts, Stellate Cells, and Matrix/Vascular Reprogramming

If injury persists, repair programs shift toward extracellular matrix deposition and architectural remodeling. Portal fibroblasts and periportal mesenchymal cells contribute to fibrosis, particularly when inflammation is concentrated in portal regions [32]. HSC and fibroblasts regulate matrix turnover through production of MMPs and TIMPs, while chronic inflammation promotes angiogenesis and vascular remodeling. These changes distort lobular perfusion and reduce the capacity for full functional restitution, even after removal of the initiating insult [33].

### 3.5. Oxidative Stress and Canonical Signaling Axes as Integrative Mechanistic Nodes

Reactive oxygen species (ROS) generated by neutrophils and macrophages during antimicrobial responses can injure hepatocytes and endothelial/stellate cells through lipid oxidation, enzyme inactivation, and DNA damage; stressed hepatocytes can further contribute to ROS generation. When antioxidant capacity is exceeded, oxidative stress also functions as a signaling amplifier by activating inflammation-associated kinases and transcription factors [34]. Mechanistically, porcine hepatic inflammation is coordinated largely through NF-κB, MAPK, and JAK/STAT. NF-κB activation follows IκB phosphorylation and degradation in response to upstream cues such as PAMPs/DAMPs, pro-inflammatory cytokines, and oxidative stress, leading to nuclear transcription of cytokines, chemokines, adhesion molecules, inflammatory effector enzymes, and survival/proliferation genes [35]. MAPK signaling integrates receptor and stress cues through extracellular signal-regulated kinases 1 and 2 (ERK1/2), *c*-Jun *N*-terminal kinases 1 and 2 (JNK1/2), and p38 branches: JNK supports AP-1 activity and stress-associated apoptosis, p38 promotes translation/production of TNF-α, IL-1β, and IL-6 and is regarded as a major pro-inflammatory kinase in hepatic inflammation, and ERK1/2 is more closely linked to proliferative repair programs [36]. The JAK/STAT pathway mediates cytokine and interferon signaling and is central to acute-phase programming and antiviral defense; IL-6–gp130 signaling activates STAT3-dependent acute-phase gene expression, interferon-STAT signaling induces antiviral states, and additional signals (including IL-22, hepatocyte growth factor, and leptin) modulate hepatocyte survival/regeneration and stellate-cell activity through STAT-linked mechanisms [36].

## 4. Curcumin—Properties and Mechanism of Action

**Figure 3:**
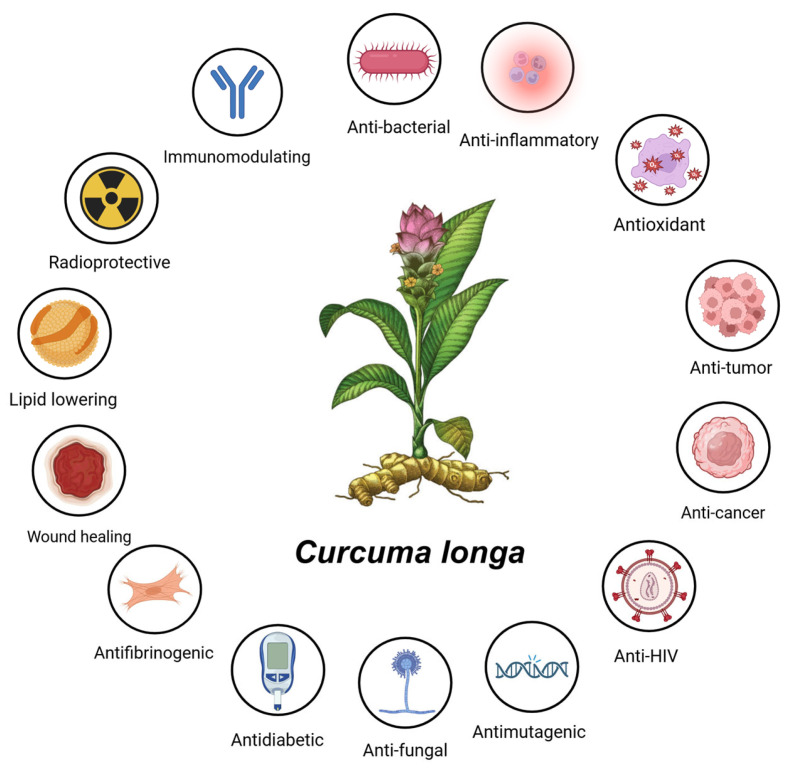
Broad spectrum of biological activities of curcumin. *Curcuma longa*, the plant source of curcumin, exhibits a wide range of experimentally confirmed biological properties. Figure has been created with www.biorender.com (accessed on 1 May 2025).

Curcumin, the principal polyphenolic constituent of *Curcuma longa* (turmeric), is widely investigated as a multi-target bioactive compound with anti-inflammatory and antioxidant actions that map onto the core biological processes driving tissue injury in inflammatory conditions, including liver pathology [36]. Mechanistically, curcumin is best framed as a network modulator: it attenuates pro-inflammatory transcription and post-translational cytokine activation while strengthening redox-adaptive cytoprotective programs. This combined profile is particularly relevant in settings where macrophage activation, cytokine amplification, oxidative stress, and downstream parenchymal cell injury occur as a coupled system. The broad spectrum of biological activities attributed to curcumin, spanning anti-inflammatory, antioxidant, antimicrobial, and cytoprotective effects, is summarized in Figure 3.

Curcumin (1,7-bis-(4-hydroxy-3-methoxyphenyl)-1,6-heptadiene-3,5-dione) [37], also known as diferuloylmethane [38], constitutes approximately 60–70% of the crude extract obtained from the rhizome of *Curcuma longa*. This extract also contains demethoxycurcumin (20–27% of the crude extract) and bisdemethoxycurcumin (10–15% of the crude extract), along with numerous other, less abundant secondary metabolites [39]. The molecular formula of curcumin is C_21_H_20_O_6_, and its molecular weight is 368.38 g/mol. The melting point of curcumin is approximately 183 °C.

Curcumin exhibits diketo/keto–enol tautomerism, which arises from the presence of a β-diketone moiety within its molecular structure [40,41]. The enol form predominates in solutions in which the solvent consists of a water/acetonitrile mixture [42]. Curcumin solutions are unstable and display an intense yellow color, which changes to dark red under alkaline conditions [43].

Furthermore, curcumin is characterized by a predominance of hydrophobic properties, resulting from the presence of nonpolar regions within the aliphatic linker, aromatic rings, and methyl groups [44]. Nevertheless, the molecule contains three hydroxyl groups that can undergo deprotonation at sufficiently high pH values, conferring a negative charge to the molecule. Consequently, under acidic and neutral conditions—where the hydroxyl groups remain protonated—curcumin retains a hydrophobic character and exhibits low water solubility. In contrast, under alkaline conditions, following deprotonation of these groups, its hydrophilicity and aqueous solubility increase [45].

### 4.1. Phytochemical Composition, Pharmacological Profile and Safety of Curcuma longa and Curcumin

To provide a broader pharmacological and translational background, the phytochemical composition, biological activity profile, and safety considerations of *Curcuma longa* and curcumin are summarized below.

Approximately 235 phytochemical compounds have been identified in the rhizome of *Curcuma* species [46]. The rhizome of *Curcuma longa* contains a wide spectrum of bioactive constituents, including alkaloids, carbohydrates, glycosides, saponins, steroids, proteins, terpenoids, flavonoids, anthraquinones, phlobotannins, and tannins, with curcuminoids—namely curcumin, demethoxycurcumin, and bisdemethoxycurcumin—representing the principal bioactive fraction. According to previous reports [47], ethanolic extracts of *Curcuma longa* rhizome exhibit notable anticancer activity, with IC_50_ values of 19.88 ± 0.50 µg/mL for prostate cancer cells (DU-145) and 11.27 ± 0.37 µg/mL for oral squamous carcinoma cells (SCC-29B), while displaying relatively low cytotoxicity toward control monkey kidney cells (Vero; IC_50_ ≈ 525 µg/mL). Interestingly, isolated curcuminoids demonstrated even higher cytotoxic activity (IC_50_ = 23.9 ± 0.45 µg/mL), indicating that both extract composition and the relative proportions of individual curcuminoids may influence biological efficacy and warrant further investigation.

Animal toxicity studies conducted in rats, guinea pigs, and monkeys suggest that oral administration of curcumin does not induce significant adverse effects under experimental conditions [48]. However, clinical studies in humans report a broader spectrum of side effects, including nausea, diarrhea, abdominal pain, headache, and elevated serum alkaline phosphatase and lactate dehydrogenase levels, particularly at higher intake ranges (500 mg–3.6 g/day) [49,50] Although turmeric and curcumin have been widely investigated for their hepatoprotective properties, rare cases of liver injury associated with high-dose supplementation have been reported, including during pregnancy. These cases were accompanied by laboratory abnormalities such as elevated ALT, ALP, GGT, and non-fasting serum bile acids following consumption of 5–10 g/day of turmeric [51].

High doses of turmeric may also impair iron absorption, and caution is warranted when curcumin is used concomitantly with anticoagulant or antiplatelet medications, as it may prolong bleeding time. Consequently, therapeutic supplementation should be avoided in individuals with bleeding disorders and discontinued at least two weeks prior to surgical procedures. Because curcumin may also influence blood glucose homeostasis, caution is advised when it is administered together with hypoglycaemic agents. The Acceptable Daily Intake (ADI) for curcumin established by the Joint FAO/WHO Expert Committee on Food Additives is 0–3 mg/kg body weight [52].

Importantly, substantial differences exist between dietary consumption of turmeric as a culinary spice and high-dose curcumin supplementation. Typical culinary use provides relatively low amounts of curcuminoids, generally well below the ADI [53], and is considered safe, whereas supplements often contain concentrated extracts or formulations designed to enhance bioavailability, such as piperine-containing or lipid-based delivery systems. These higher-dose formulations may increase the risk of gastrointestinal discomfort, liver enzyme alterations, and drug–nutrient interactions, highlighting the need for careful dose selection and monitoring when curcumin is used for therapeutic purposes.

### 4.2. Transcriptional Suppression of Inflammatory Programs (NF-κB, AP-1) and Mediator “Outputs”

A central mechanism attributed to curcumin is inhibition of NF-κB activation, leading to reduced transcription of pro-inflammatory genes and lower production of key cytokines such as TNF-α, IL-1β, and IL-6 [36]. Importantly, NF-κB inhibition is not limited to lowering cytokines; it also plausibly reduces the broader inflammatory output set that sustains tissue inflammation, including chemotactic signaling and inducible enzymes that generate reactive intermediates and lipid mediators. Curcumin is also reported to modulate activator protein-1 (AP-1) and MAPK signaling, thereby further constraining stress- and cytokine-driven gene expression and decreasing the likelihood of sustained inflammatory escalation [36]. Together, NF-κB/AP-1/MAPK modulation provides a coherent upstream explanation for reductions in multiple downstream inflammatory readouts rather than isolated single-mediator effects.

A practical and repeatedly emphasized downstream consequence is reduced expression of inducible inflammatory enzymes, particularly inducible nitric oxide synthase (iNOS) and cyclooxygenase-2 (COX-2), which amplify inflammation through nitric oxide–related reactive species and eicosanoid signaling. Consistent with this, curcumin has been reported to inhibit COX-2 and 5-lipoxygenase (5-LOX), key enzymes in eicosanoid synthesis, thereby reducing formation of prostaglandins (PGE_2_) and leukotrienes and limiting accumulation of lipid-derived inflammatory signals that reinforce vascular activation and leukocyte recruitment [36].

### 4.3. NLRP3 Inflammasome Inhibition as Control of Cytokine Maturation (Not Only Transcription)

To date, direct assessment of NLRP3 inflammasome activation in porcine liver tissue following curcumin administration has not been reported. Accordingly, references to NLRP3 modulation in pigs are presented as mechanistic inference based on macrophage-based and non-porcine liver models, rather than as experimentally validated porcine evidence.

Curcumin has been reported to limit activation of the NOD-like receptor family, pyrin domain-containing 3 (NLRP3) inflammasome, resulting in reduced secretion of mature IL-1β [54]. This is mechanistically important because inflammasome activity operates as a post-translational amplification node: even when upstream inflammatory transcription is partially attenuated, inflammasome-dependent maturation of IL-1β can sustain a high-inflammatory state. Therefore, simultaneous dampening of NF-κB–linked transcriptional tone and NLRP3-linked cytokine maturation provides a plausible basis for curcumin’s reported ability to reduce both the intensity and persistence of innate inflammatory cascades [54].

### 4.4. Immunomodulation: Macrophage Phenotype Tuning and IL-18 Suppression

Curcumin has also been described as an immunomodulator capable of reshaping innate immune effector phenotypes. In macrophage populations, curcumin reportedly suppresses excessive M1-like pro-inflammatory activity and promotes reprogramming toward an M2-like phenotype, a shift commonly associated with reduced release of pro-inflammatory mediators and lower tissue-destructive signaling [55]. Mechanistically, this point matters because macrophages can function as sustained sources of cytokines, reactive intermediates, and remodeling signals during chronic inflammatory states.

Yadav et al. reported that curcumin inhibited the release of IL-18 from lipopolysaccharide-stimulated macrophages in a concentration-dependent manner without cytotoxicity [36]. Given the pro-inflammatory role of IL-18 as an amplifier of inflammatory signaling, suppression of IL-18 provides a plausible macrophage-centered pathway complementary to NF-κB/AP-1 inhibition [36]. Human observations support systemic anti-inflammatory plausibility: in a pilot rheumatoid arthritis trial, curcumin at 500 mg/day reduced disease activity and lowered inflammatory markers (ESR, CRP), with reported improvement exceeding standard anti-inflammatory treatment in that study context [56]. While not liver-specific, this clinical signal supports the concept that curcumin can meaningfully shift measurable inflammatory burden in vivo.

### 4.5. Antioxidant Function: Direct ROS/RNS Control Plus Nrf2/ARE-Driven Cytoprotection

Curcumin is widely characterized as an antioxidant and regulator of cellular redox homeostasis. It has been reported to directly scavenge reactive oxygen species (ROS) and reactive nitrogen species (RNS), limiting lipid peroxidation and oxidative damage to cellular macromolecules. In oxidative-stress settings, curcumin reduces accumulation of lipid peroxidation products such as malondialdehyde (MDA) and supports endogenous antioxidant capacity; for example, in a brain ischemia model, curcumin was reported to reduce lipid peroxidation and restore endogenous antioxidant defenses, including glutathione and antioxidant enzyme activity [54].

Critically, antioxidant effects are not restricted to direct radical scavenging. Curcumin has been reported to activate the nuclear factor erythroid 2–related factor 2/antioxidant response element (Nrf2/ARE) pathway, increasing expression of cytoprotective enzymes such as heme oxygenase-1 (HO-1), NAD(P)H:quinone oxidoreductase 1 (NQO1), and glutathione transferases [56]. This mechanism is highly valuable in injury biology because it increases the capacity for detoxification and redox buffering, rather than providing only transient scavenging. In inflammatory contexts where oxidative stress amplifies inflammatory signaling and propagates tissue injury, Nrf2/ARE induction provides a mechanistically coherent basis for sustained cytoprotection [54,56].

### 4.6. Mechanistic Synthesis and Relevance to Hepatoprotection Logic

Taken together, curcumin’s reported effects—(i) suppression of NF-κB/AP-1/MAPK-driven inflammatory transcription, (ii) inhibition of inflammasome-dependent IL-1β maturation (NLRP3), (iii) modulation of macrophage inflammatory phenotype and IL-18 output, and (iv) reinforcement of antioxidant defenses via Nrf2/ARE—form an internally consistent mechanistic package that targets both inflammatory initiation/amplification and oxidative injury propagation [36,54,55,56,57]. These nodes align directly with the convergent mechanisms underlying hepatic inflammation and injury, providing a rational mechanistic foundation for evaluating curcumin as an adjunct candidate in liver dysfunction contexts.

## 5. Problem with Curcumin Bioavailability

Curcumin, despite its widely documented biological activity, exhibits very low oral bioavailability, meaning that only a small fraction of an ingested dose reaches systemic circulation as pharmacologically active parent compound. This limitation reflects a multi-step absorption–distribution–metabolism–excretion (ADME) bottleneck in which poor gastrointestinal dissolution restricts uptake, and the absorbed fraction is rapidly metabolized and cleared. Consequently, plasma exposure to free (unconjugated) curcumin is typically low and short-lived after oral administration, which directly constrains dose–exposure–effect interpretation and is a central determinant of translational plausibility.

A primary barrier is curcumin’s strong hydrophobicity and practical insolubility in water, which limits dissolution in intestinal fluids and therefore the fraction available for epithelial absorption. For poorly soluble compounds, absorption is often dissolution-limited: if the compound does not enter solution (or a solubilized colloidal phase), it cannot be efficiently transported across the intestinal barrier. Even when some absorption occurs, curcumin is subject to pronounced first-pass metabolism in enterocytes and hepatocytes, dominated by phase II conjugation (glucuronidation and sulfation). The resulting metabolites—curcumin-glucuronide and curcumin-sulfate—are commonly described as having markedly lower biological activity than the parent compound, shifting systemic exposure away from free curcumin. In parallel, rapid elimination of parent compound and metabolites contributes to a short apparent half-life and low sustained systemic exposure over time.

Curcumin is generally recognized as a safe, food-derived bioactive compound with minimal toxicity. Nevertheless, its practical use is constrained by several pharmacological limitations, primarily arising from low systemic bioavailability. These factors underlie pronounced species-specific differences in pharmacokinetics and dose-dependent effects, which complicate dose extrapolation from rodent models to livestock species such as pigs. Therefore, species-specific pharmacokinetic and toxicological considerations are essential when interpreting curcumin supplementation strategies in pigs. In rodents, oral dosing often results in detectable, albeit low, plasma levels of curcumin and its metabolites, with a short elimination of half-life and modest tissue distribution of parent compound relative to reduced forms. In contrast, human pharmacokinetic studies consistently report very low or undetectable levels of free curcumin in plasma after oral administration [58]. Although direct comparative pharmacokinetic studies in pigs are limited, available evidence from piglet models reinforces the principle that oral curcumin exhibits low systemic bioavailability in monogastric livestock, similar to humans. Curcumin’s hydrophobic nature and rapid metabolic clearance limit its systemic exposure after administration. In pigs, hepatic and intestinal metabolism substantially reduce free curcumin levels, resulting in low circulating concentrations and predominant local gastrointestinal effects rather than robust systemic action when administered in feed additives [59]. Formulations such as nano-encapsulated curcumin enhance solubility and potential bioactivity.

In humans, single dose of 12 g has been administered without serious adverse effects, although gastrointestinal discomfort has been reported at high intake levels [60]. Prolonged administration of high doses of curcumin also did not cause side effects. Clinical studies assessing the therapeutic use of turmeric or curcumin in the management of arthritis or postoperative inflammation reported that daily curcumin intakes ranging from 1.2 to 2.1 g, administered over periods of 2 to 6 weeks, were not associated with any adverse effects [61,62]. Regulatory evaluations, such as those by the European Food Safety Authority (EFSA), have established an acceptable daily intake (ADI) of 3 mg/kg body weight per day for curcumin when used as a food additive [63]. In a 90-day subchronic toxicity study in rats, administration of high curcumin doses (100 mg per kg of weight per day) resulted in metabolic imbalance, oxidative stress, and altered liver function, suggesting that curcumin exhibits dose-dependent toxicity beyond a defined safety threshold [64]. Similarly, toxicological evaluations of curcumin-loaded nanocomplexes in mice and hamsters demonstrated that while low and moderate doses were well tolerated, chronic exposure to high doses led to histopathological changes and alterations in biochemical parameters [65]. In pigs, available data suggest that curcumin is generally well tolerated when administered at nutritionally relevant doses, although the safety margin remains insufficiently defined. Dietary supplementation studies in finishing pigs for forty days using curcumin nanospheres in doses ranging from 150 to 250 mg of curcumin over periods of several weeks did not report adverse effects on growth performance, serum biochemical indices, gastrointestinal health, or meat quality when curcumin was included at low to moderate dietary levels [66]. Moreover, in pigs with intrauterine growth retardation, dietary curcumin supplementation administered for several weeks at a dose of 400 mg per kg of weight exerted beneficial effects on intestinal oxidative status without negatively affecting growth performance, indicating a favorable local intestinal response at the applied dose range [59].

Pharmacokinetic constraints have direct implications for interpreting biological effects. Many in vitro mechanisms are reported at concentrations that are difficult to achieve systemically with unformulated oral curcumin. Therefore, biological outcomes should be interpreted in a formulation- and exposure-aware manner, and studies using enhanced-delivery systems should not be treated as directly comparable to studies using plain curcumin unless exposure equivalence is demonstrated. Importantly, low systemic exposure to parent curcumin does not exclude biological relevance: some effects may plausibly arise through local intestinal activity and downstream gut–liver axis signaling even when circulating free curcumin remains low. For hepatoprotection claims, the key requirement is to align mechanistic conclusions with achievable exposure in the relevant administration context.

Several strategies have been developed to improve curcumin exposure, each addressing a different component of the ADME bottleneck. Piperine, an alkaloid from black pepper, is among the most used bioenhancers; it increases curcumin exposure by inhibiting enzymes involved in glucuronidation (e.g., UDP-glucuronosyltransferases) and by improving intestinal absorption. In some studies, co-administration with piperine increased curcumin bioavailability by up to approximately 2000%, although the magnitude is dependent on dose, formulation, and study conditions. Because curcumin is fat-soluble, co-administration with dietary lipids can enhance solubilization in the intestinal lumen and improve uptake. More advanced formulation strategies—such as liposomes, emulsions, and phospholipid complexes (e.g., curcumin–phosphatidylcholine)—can further increase apparent bioavailability by improving solubility, stabilizing curcumin in the gastrointestinal environment, facilitating intestinal transport, and partially mitigating first-pass loss. For example, a curcumin formulation with soy lecithin produced 29-fold higher plasma concentrations than curcumin alone [36].

For porcine applications, formulation and feeding matrix are likely to be particularly influential because oral administration frequently occurs via feed, where diet fat content, meal timing, and gastrointestinal transit can vary across production settings. Species differences in metabolic capacity and conjugation patterns may also influence the relative abundance of parent compound versus conjugated metabolites, limiting direct extrapolation from non-porcine pharmacokinetic profiles without supporting exposure measurements. Accordingly, mechanistically credible interpretation across studies requires consistent reporting of curcumin identity and composition, formulation class, dose (mg/kg), route, dosing duration and timing relative to injury induction, feeding matrix (including lipid content for oral studies), and—where feasible—evidence of systemic or tissue exposure (e.g., plasma/tissue detection or pharmacokinetic parameters). Importantly, findings obtained using enhanced-delivery systems (e.g., phospholipid complexes, nano formulations, or bioenhancers) should not be directly extrapolated to conventional curcumin preparations, as these formulations differ substantially in absorption, systemic exposure, and pharmacokinetic behavior.

In pigs, curcumin is typically administered orally as a feed supplement, and direct pharmacokinetic data describing plasma or hepatic exposure remain scarce. Reported in vivo pig studies therefore rely primarily on administered dose, formulation class, and downstream hepatic endpoints rather than measured tissue concentrations. This limits direct dose–exposure–effect linkage and necessitates cautious interpretation of pathway-level mechanisms, particularly those derived from in vitro systems requiring higher concentrations. Nevertheless, biologically relevant hepatic effects may plausibly arise through local intestinal activity and gut–liver axis signaling even when circulating free curcumin remains low. To align mechanistic interpretation with achievable exposure in pigs, a structured summary of porcine in vivo studies, including dose, formulation, duration, hepatic endpoints, and reported exposure data, is provided in Table 3.

Where applicable, mechanistic interpretations reflect formulation-specific contexts, and effects observed with enhanced-delivery systems should not be generalized to conventional curcumin preparations without exposure equivalence.

## 6. Mechanisms of Action of Curcumin in Liver Dysfunctions

**Figure 4:**
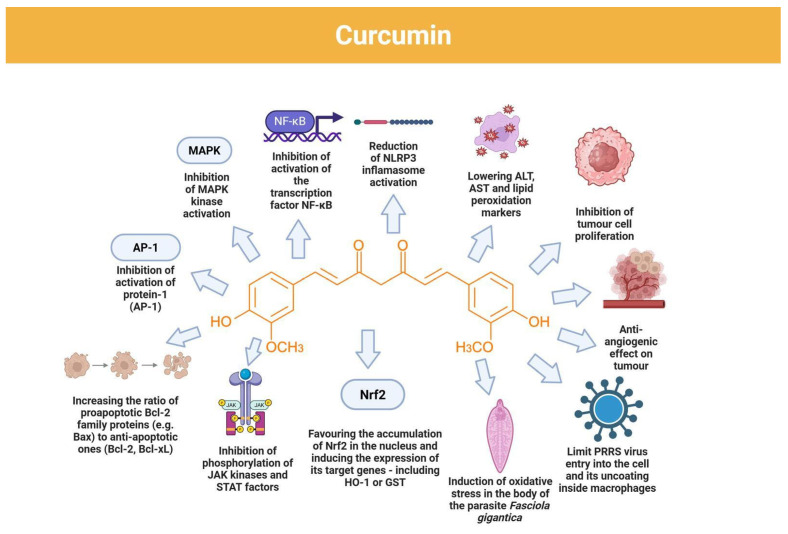
Molecular mechanisms underlying the biological activity of curcumin. Curcumin exerts its hepatoprotective, anti-inflammatory, and anti-tumor effects through the modulation of multiple cellular signaling pathways. Figure has been created with www.biorender.com (accessed on 1 May 2025).

Curcumin exerts hepatoprotective effects through a coordinated, multi-node mechanism that targets the coupled processes driving liver dysfunction: (i) dampening of innate-inflammatory signal transduction and cytokine amplification, (ii) reinforcement of antioxidant and detoxification capacity, (iii) normalization of lipid-handling programs under inflammatory stress, and (iv) modulation of hepatocyte survival–death balance. Functionally, these actions converge on reduced hepatocellular injury (lower leakage enzymes), reduced oxidative damage (lower lipid/protein oxidation), improved metabolic stability (less pathological lipid accumulation), and attenuation of macrophage/Kupffer-cell–driven inflammatory escalation—mechanistic features that are particularly relevant in toxin-, endotoxin-, and infection-associated hepatic injury phenotypes. The principal molecular targets and pathway interactions discussed below are summarized in Figure 4.

Collectively, these distinctions underscore that antioxidant/Nrf2-linked mechanisms currently represent the most robust porcine-validated pathway, whereas inflammatory signaling pathways (NF-κB, NLRP3) remain supported primarily by indirect or extrapolated evidence.

### 6.1. Protection Against Toxin-Induced Liver Injury: Redox Stabilization and Limitation of Injury Propagation

**Figure 5:**
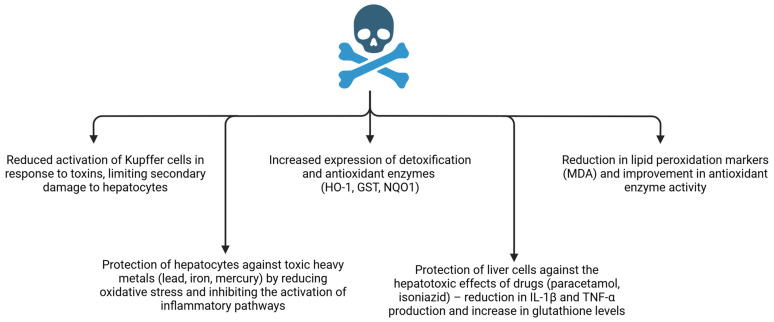
Hepatoprotective mechanisms involved in the liver’s response to toxic agents. Exposure to toxic substances activates inflammatory and oxidative pathways in the liver, leading to hepatocyte damage. Curcumin attenuates toxin-induced injury by various mechanisms. Figure has been created with www.biorender.com (accessed on 1 May 2025).

In toxin-driven liver injury, curcumin consistently reduces oxidative stress and limits hepatocyte damage. In the carbon tetrachloride (CCl_4_) model of acute hepatic injury, curcumin reduced parenchymal injury and improved biochemical indices of liver damage. CCl_4_ hepatotoxicity is accompanied by increased activity of liver enzymes (transaminases, ALP, γ-GTP) and depletion of antioxidant reserves (glutathione, vitamins C and E). Curcumin attenuated these changes by lowering ALT and AST, reducing lipid peroxidation markers, and restoring hepatic glutathione and antioxidant vitamin content [36]. Mechanistically, this profile supports preservation of membrane integrity and intracellular redox buffering, which limits secondary amplification loops in which oxidative injury propagates inflammatory activation and further parenchymal damage. The major toxicant-induced injury processes and the principal points of curcumin intervention are schematized in Figure 5.

### 6.2. Anticancer Actions Relevant to Hepatic Malignancy Biology: Survival Signaling Blockade, Apoptosis Induction, and Angiogenesis Restraint

Curcumin demonstrates anticancer activity in multiple tumor models and cell systems, including mechanisms relevant to hepatic malignancy biology: suppression of proliferative/survival signaling, induction of apoptosis, and anti-angiogenic effects. At the pathway level, curcumin inhibits growth- and survival-associated axes such as phosphoinositide 3-kinase/protein kinase B (PKB)/mechanistic (mammalian) target of rapamycin (PI3K/Akt/mTOR) and MAPK signaling [36]. Pro-apoptotic effects involve mitochondrial apoptosis programming, including an increased ratio of pro-apoptotic Bcl-2 family proteins (e.g., Bax) relative to anti-apoptotic proteins (Bcl-2, Bcl-xL) and activation of caspase cascades. For example, in melanoma cells, curcumin induced cytochrome c release and activation of caspase-9 and caspase-3, culminating in apoptosis [36]. Curcumin also exhibits anti-angiogenic activity by suppressing pro-angiogenic gene programs and inflammation-dependent mediators that support neovascularization and tumor progression [36]. Although not species-specific, these mechanisms map onto conserved control points in tumor biology—cell-cycle progression, apoptosis resistance, and vascular remodeling—that are also relevant to hepatic oncogenesis.

### 6.3. Anti-Inflammatory Signaling: NF-κB Inhibition as an Upstream Controller of Cytokines and Inducible Inflammatory Effector Programs

Direct experimental validation of NF-κB pathway modulation by curcumin in porcine liver tissue remains limited. Therefore, NF-κB-centered interpretations in pigs are primarily inferred from conserved inflammatory signaling demonstrated in non-porcine mammalian liver models, unless explicitly measured in porcine studies.

A core anti-inflammatory mechanism of curcumin is inhibition of NF-κB, which reduces transcription of pro-inflammatory mediators and downstream cytokine output. In BV2 microglia, curcumin modulated Toll-like receptor 4 (TLR4) signaling, inhibited NF-κB activation, promoted polarization toward an anti-inflammatory M2 phenotype, and reduced secretion of TNF-α and IL-6 [NO_PRINTED_FORM]. While non-hepatic, this model is mechanistically informative because TLR4–NF-κB coupling is a conserved innate-inflammatory axis central to endotoxin-driven hepatic inflammation and Kupffer-cell activation. In vivo corroboration is also reported outside the liver: in a rat model of chronic smoke-induced lung inflammation (COPD), oral curcumin (100 mg/kg) inhibited excessive NF-κB activation and decreased TNF-α and IL-6 while increasing PPAR-γ activity, consistent with suppression of inflammatory transcriptional tone and/or reinforcement of counter-regulatory anti-inflammatory signaling [67]. Together, these data support NF-κB as a high-leverage node through which curcumin can lower inflammatory “gain,” thereby limiting cytokine-driven tissue injury and downstream inflammatory effector programs.

### 6.4. MAPK Modulation: Reducing Stress-Integrated Inflammatory Mediator Production and Nitric Oxide Signaling

Curcumin modulates MAPK signaling, which integrates cellular stress cues and cytokine receptor signals and thereby regulates inflammatory mediator synthesis and tissue injury severity. In LPS-stimulated BV2 microglia, curcumin inhibited activation of p38 and ERK1/2, accompanied by reduced nitric oxide production and decreased pro-inflammatory cytokine output [68]. Similar pathway-level effects have been reported in vivo: in DSS-induced experimental colitis in mice, curcumin was associated with reduced p38 phosphorylation and decreased mucosal cytokine expression (including IL-1β and TNF-α) [69]. Ex vivo, curcumin inhibited p38 MAPK activation in intestinal biopsies from patients with inflammatory bowel disease and concurrently increased IL-10, consistent with a shift toward a less inflammatory cytokine profile [70]. Mechanistically, these findings position p38-centered MAPK restraint as a plausible route by which curcumin reduces stress-amplified mediator production (including nitric oxide-related pathways), thereby limiting collateral tissue damage.

### 6.5. JAK/STAT Attenuation via SOCS Induction: Strengthening Endogenous Negative Feedback in Cytokine Signaling

Curcumin can inhibit phosphorylation of JAK kinases and STAT transcription factors, thereby limiting pro-inflammatory signaling through the JAK/STAT axis [71]. This effect is frequently linked to induction of endogenous negative regulators, particularly SOCS1 and SOCS3, which provide feedback control of cytokine receptor signaling and prevent prolonged cytokine signaling states [72,73]. In BV2 microglia, Porro et al. reported that curcumin increased SOCS-1 expression and reduced phosphorylation of JAK2 and STAT3 in LPS-stimulated cells, shifting cytokine output toward increased IL-10 and IL-4 with reduced pro-inflammatory mediators [74]. Similar regulatory effects were reported in dendritic cells, where curcumin inhibited maturation and activation via JAK/STAT/SOCS signaling, reducing antigen presentation and attenuating inflammatory responses in an intestinal inflammation model [75]. In TNBS-induced colitis in rats, dietary curcumin increased SOCS-1 and inhibited STAT1 phosphorylation, reduced excessive cytokine production (including TNF-α and IL-6), and improved histological outcomes; importantly, inhibition of SOCS-1 abolished curcumin’s benefit, supporting SOCS-1–dependent restraint of JAK/STAT signaling as a functional mechanism in that model [76]. Collectively, these findings suggest that curcumin can reduce inflammatory signaling not only by suppressing upstream drivers but also by reinforcing intrinsic “braking” circuitry that constrains cytokine amplification.

### 6.6. Antioxidant Pathway Activation: Nrf2-Driven Cytoprotection Plus Suppression of Upstream ROS Generation

In contrast to inflammatory signaling pathways, activation of Nrf2-centered antioxidant responses has been directly demonstrated in porcine models, including in vivo upregulation of Nrf2 target genes such as HO-1, GST, and NQO1 under conditions of oxidative and endotoxin-associated hepatic stress.

Curcumin activates intracellular antioxidant programs, with Nrf2 as a central transcriptional target. Curcumin promotes Nrf2 nuclear accumulation and induces expression of cytoprotective genes, including heme oxygenase-1 (HO-1) and glutathione S-transferase (GST) [77]. This is mechanistically relevant because oxidative stress both causes direct hepatocyte damage and amplifies inflammatory signaling, creating a coupled injury loop. Beyond transcriptional induction of antioxidant defenses, curcumin can reduce upstream ROS production: in equine neutrophils, Derochette et al. showed that curcumin inhibited the NADPH oxidase complex, reducing superoxide production and thereby weakening ROS-driven amplification and collateral tissue injury [78]. In vivo findings are consistent: in the TNBS colitis model, curcumin reduced oxidative stress markers such as MDA and increased antioxidant enzyme activity in colon tissue [76]. Together, these results support a dual antioxidant mechanism—lower ROS generation plus increased enzymatic detoxification/redox buffering—that is directly relevant to hepatocyte protection in oxidative and inflammatory injury states. Importantly, shifts in antioxidant or redox biomarkers alone should not be interpreted as evidence of functional liver recovery unless accompanied by improvements in leakage enzymes, histopathological architecture, or other model-relevant functional endpoints.

### 6.7. Evidence in Pigs: Redox Protection, Antiviral Mechanisms, Endotoxin-Associated Hepatic Injury, Lipid Handling, and Epitranscriptomic Regulation

**Figure 6:**
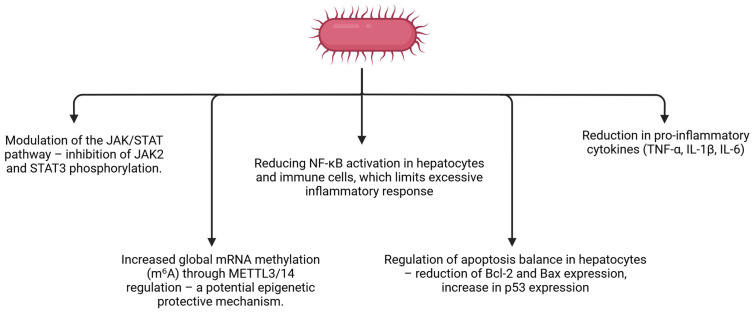
Key molecular mechanisms involved in the hepatic response to bacterial infection. Bacterial pathogens activate multiple intracellular signaling pathways in hepatocytes and immune cells, leading to inflammation and tissue injury. Figure has been created with www.biorender.com (accessed on 1 May 2025).

Pig-specific models provide direct evidence that curcumin modulates oxidative stress and hepatic injury pathways in vivo, strengthening translational relevance for swine liver dysfunction. Key response nodes activated during infection- and endotoxin-driven hepatic inflammation (and their modulation by curcumin-relevant pathways) are summarized in Figure 6.

In a pro-oxidative stress model associated with intrauterine growth restriction (IUGR), supplementation with 200 mg/kg curcumin reduced lipid peroxidation markers (malondialdehyde, carbonyl proteins) and increased antioxidant enzyme activities (catalase, peroxidase, SOD). Curcumin also increased expression of Nrf2-dependent antioxidant genes in liver and other tissues, including HO-1, GST, and NQO1, consistent with enhanced antioxidant capacity and improved redox status in vivo [79].

**Figure 7:**
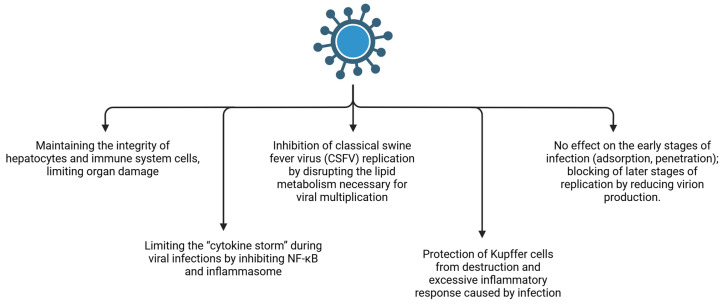
Antiviral mechanisms supporting hepatic protection during viral infections in swine. Viral infections in pigs trigger cellular injury and excessive inflammation in the liver. Curcumin helps maintain the structural integrity of hepatocytes and immune cells, thereby reducing organ damage. Figure has been created with www.biorender.com (accessed on 1 May 2025).

Curcumin also exhibits antiviral activity against pathogens of major relevance to swine health, with mechanisms that intersect with lipid metabolism and macrophage biology. Yi and Zhou reported that curcumin inhibited replication of classical swine fever virus (CSFV) in vitro, primarily by interfering with lipid metabolism required for later stages of viral replication; curcumin did not affect viral adsorption/entry but impaired subsequent replication steps and reduced production of viral particles [80]. Curcumin has also been described to inhibit Porcine Reproductive and Respiratory Syndrome Virus (PRRSV) infection by impeding fusion and uncoating within host cells. In vitro work showed that curcumin strongly inhibited PRRS virus infection in both MARC-145 cells and primary porcine alveolar macrophages, with viral load reductions exceeding 90% compared with controls [81]. The proposed antiviral mechanisms relevant to swine infection control and downstream hepatic protection are summarized in Figure 7.

In endotoxin-associated liver injury, curcumin demonstrates protective effects in piglets exposed to LPS. Supplementation reduced LPS-induced elevations of cytolytic enzymes (AST, lactate dehydrogenase (LDH)) in blood and liver and prevented lipid accumulation in hepatocytes by lowering LPS-elevated hepatic cholesterol and triglycerides (*p* < 0.05). At the molecular level, curcumin reversed endotoxin-induced gene-expression disturbances by inhibiting induction of lipogenic regulators Sterol Regulatory Element-Binding Protein-1c (SREBP-1c) and Stea-royl-CoA desaturase-1 (SCD-1) and modulating apoptosis-related genes (reduced Bcl-2 and Bax; increased p53 mRNA), consistent with normalization of lipid handling and hepatocyte survival–death balance. Notably, curcumin increased global mRNA methylation (m6A) in liver by regulating expression of methyltransferases (METTL3/14) and demethylases, suggesting that epitranscriptomic regulation of RNA fate contributes to its protective actions in endotoxin-associated hepatic injury [82]. Given the heterogeneous availability of porcine data across mechanistic domains, Table 4 provides a structured comparison of porcine in vivo evidence versus non-porcine liver models, indicating where curcumin-mediated effects are directly supported in pigs and where conclusions remain extrapolated.

### 6.8. Additional Anti-Infective and Detoxification-Relevant Actions: Parasite Viability and Chemical Injury Mitigation

Curcumin has demonstrated direct antiparasitic activity in vitro; for example, it induced death of adult liver flukes (*Fasciola gigantica*) by promoting oxidative stress within the parasite. Curcumin reduced parasite antioxidant defenses (lower glutathione, SOD activity, glutathione transferase) and intensified damage to tegument and proteins, compromising detoxification capacity and viability [83]. Curcumin has also been reported to attenuate liver injury induced by environmental pollutants such as polybrominated biphenyls via Nrf2 pathway activation, increasing expression of phase II detoxification and antioxidant enzymes and limiting lipid peroxidation in hepatocytes. Additional studies indicate protection against heavy metals (e.g., lead, iron, mercury) and hepatotoxic drugs through suppression of oxidative stress and inhibition of inflammatory signaling [84]. These observations reinforce curcumin’s relevance to hepatic injury contexts where xenobiotic burden and oxidative stress are dominant contributors and where hepatoprotection plausibly depends on strengthening detoxification and redox buffering programs.

### 6.9. Kupffer Cells and Macrophage Control: Phenotype Modulation, Safety Signals in Pigs, and Implications for Viral and Toxic Injury

Because Kupffer cells are central amplifiers of hepatic inflammation, curcumin’s effects on macrophage phenotype and cytokine output are mechanistically decisive for liver protection. Curcumin is considered safe in pigs and does not appear to impair baseline Kupffer-cell function under physiological conditions. Instead, evidence suggests it can shift macrophage/Kupffer phenotypes toward a less pro-inflammatory state by inhibiting constitutive NF-κB activity and reducing production of TNF-α, IL-1β, and IL-6 in response to stimuli [85]. Safety studies did not show Kupffer-cell damage or dysfunction in pigs receiving chronic curcumin, and no inflammatory infiltrates or pathological liver-histology changes were observed [86]. In viral contexts, suppression of PRRSV infection in porcine macrophages provides a mechanistic basis for preserving macrophage function and limiting infection-driven cytokine escalation [81]. In acute chemical injury contexts (e.g., iron overload or hepatotoxic drug exposure), curcumin has been reported to reduce Kupffer-cell cytokine secretion (including IL-1β and TNF-α) and improve antioxidant capacity (e.g., increased glutathione), consistent with reduced macrophage-driven inflammatory amplification and mitigation of downstream hepatocyte injury via NF-κB-linked mechanisms [87].

### 6.10. Curcumin in Chronic Liver Disease Progression: Stage-Specific Mechanisms and Systemic Metabolic Integration

#### 6.10.1. Conceptual Framework: Chronic Liver Disease as a Coupled Metabolic–Inflammatory Continuum

Chronic liver disease should be conceptualized as a dynamic, multiscale continuum in which metabolic dysregulation, immune activation, and tissue remodeling evolve in a tightly coupled manner rather than as discrete pathological entities [88,89,90]. In pigs, this continuum is shaped predominantly by intersecting pressures such as dietary composition and meta-inflammation with endotoxemia [91], bacterial endotoxin-mediated hepatic injury [92], and chronic viral hepatitis models that contribute to progressive hepatic dysfunction, rather than by isolated lifestyle-related risk factors typical of human disease. These drivers converge on conserved regulatory architectures that integrate lipid flux, inflammatory signal amplification, redox balance, and cellular stress responses characteristic of chronic liver pathobiology [88,89,90].

Within this framework, curcumin cannot be meaningfully interpreted as a disease-specific intervention (e.g., “anti-NAFLD” or “antifibrotic” agent). Instead, its biological relevance emerges from its capacity to modulate shared regulatory nodes that determine whether hepatic injury remains adaptive and reversible or progresses toward structural and functional failure. This system-level interpretation is particularly appropriate for porcine liver dysfunction, where metabolic, infectious, and toxic insults frequently coexist and reinforce one another.

#### 6.10.2. Early-Stage Dysfunction (NAFLD-like Phenotypes): Immunometabolic Regulation of Hepatic Lipid Flux

In early NAFLD-like states, hepatic dysfunction is dominated by dysregulated lipid flux rather than overt inflammatory pathology, with imbalances between lipid uptake, processing, β-oxidation, and export contributing to excessive hepatic triglyceride accumulation and steatosis [93,94]. In pigs, experimental high-fat and cholesterol diet models lead to pronounced hepatic steatosis characterized by excessive lipid deposition and micro vesicular fat accumulation, indicating that lipid excess can precede significant inflammation in large animal models of NAFLD [95]. Under these conditions, lipid accumulation is not metabolically inert; instead, it generates lipotoxic intermediates that impair mitochondrial function, increase oxidative burden, and lower hepatocyte tolerance to secondary inflammatory stress, with organellar dysfunction including mitochondrial impairment contributing to disease progression [96].

Curcumin’s mechanistic relevance at this stage lies in indirect stabilization of hepatic lipid handling through modulation of the inflammatory and redox environment that governs metabolic gene regulation. By constraining oxidative pressure and inflammatory signaling intensity, curcumin limits maladaptive activation of lipid-accumulating programs and reduces peroxidative damage to membrane lipids and organelles. This immunometabolic mode of action distinguishes curcumin from classical lipid-lowering strategies and is particularly relevant in pigs, where hepatic steatosis often arises as a consequence of systemic inflammatory stress rather than primary caloric overload.

#### 6.10.3. Transition to Steatohepatitis (NASH-like Pathology): Inflammatory Gain Control and Hepatocyte Injury Thresholds

The transition from steatosis to steatohepatitis represents a qualitative shift in disease biology, marked by immune-cell recruitment, amplification of cytokine signaling, and initiation of hepatocyte injury programs [97]. In pigs, this transition is frequently driven by systemic inflammatory stimuli, including bacterial endotoxins and viral infections, rather than by metabolic stress in isolation. Kupffer-cell activation and cytokine amplification act as central drivers, creating an inflammatory microenvironment that exceeds hepatocyte adaptive capacity [98,99].

In this context, curcumin’s biological value is best understood as regulation of inflammatory signal gain rather than suppression of immune competence. By attenuating excessive amplification of inflammatory cascades, curcumin plausibly increases the threshold at which hepatocytes transition from adaptive stress responses to irreversible injury. This selective dampening of inflammatory escalation is critical in veterinary contexts, where preservation of antimicrobial and antiviral immunity is essential. Mechanistically, curcumin therefore functions as a regulator of inflammatory intensity and duration, limiting collateral tissue damage without abolishing host defense.

#### 6.10.4. Fibrogenic Progression: Disruption of Inflammatory–Remodeling Coupling

Fibrosis represents a shift from predominantly inflammatory pathology toward tissue remodeling driven by sustained mesenchymal activation and extracellular matrix deposition [97,98]. In chronic liver injury, persistent inflammatory and oxidative stress promote activation of matrix-producing cell populations, especially hepatic stellate cells, leading to excessive extracellular matrix accumulation [97]. This ongoing matrix deposition progressively distorts hepatic architecture and impairs normal liver function, reflecting the dynamic wound-healing nature of fibrogenesis rather than a static pathological state [98].

Curcumin’s relevance at this stage should be interpreted as indirect modulation of the inflammatory–fibrogenic interface rather than as direct antifibrotic activity. By reducing upstream inflammatory pressure and oxidative stress, curcumin diminishes the signaling milieu that favors continued matrix deposition and fibrogenic persistence. This mode of action is consistent with experimental observations indicating reduced fibrogenic signaling under conditions of controlled inflammation. However, it must be emphasized that curcumin is unlikely to reverse established fibrosis; its potential utility lies in slowing progression when inflammatory drivers remain active.

#### 6.10.5. Advanced Disease and Cirrhosis: Limits of Intervention and Supportive Stabilization

Cirrhosis represents the terminal stage of chronic liver disease, characterized by irreversible architectural distortion, vascular remodeling, and loss of metabolic reserve. At this stage, therapeutic strategies are inherently supportive, aiming to reduce secondary injury, prevent decompensation, and preserve residual function rather than restore normal structure [99].

From a mechanistic standpoint, curcumin’s potential role in cirrhosis is necessarily constrained. By limiting ongoing inflammatory and oxidative stress, curcumin may mitigate episodic injury triggered by infections, endotoxemia, or metabolic disturbances that commonly precipitate decompensation in advanced disease. However, the absence of dedicated porcine studies addressing cirrhosis-specific outcomes necessitates a conservative interpretation. Any proposed benefit should be framed strictly as adjunctive stabilization rather than disease modification.

#### 6.10.6. Gut–Liver Axis: Intestinal Barrier Integrity as a Determinant of Hepatic Inflammatory Load

The gut–liver axis plays a central role in chronic liver diseases, with disruption of the intestinal barrier permitting translocation of microbial products such as endotoxin into the portal circulation and directly exposing the liver to pro-inflammatory stimuli that exacerbate hepatic inflammation and injury [100,101]. Increased intestinal permeability driven by dysbiosis and barrier dysfunction facilitates the influx of bacterial components that activate hepatic innate immune responses, contributing to progression of liver pathology through sustained cytokine and immune signaling [100].

Curcumin’s reported capacity to support intestinal barrier integrity and attenuate endotoxin-associated inflammatory signaling provides a plausible indirect mechanism for reducing hepatic inflammatory burden. Notably, this gut-centered mode of action does not require high systemic concentrations of parent compound, aligning with curcumin’s pharmacokinetic constraints. By lowering the magnitude of portal inflammatory inputs, curcumin may indirectly stabilize hepatic immune activation and slow progression of liver pathology.

#### 6.10.7. Gut–Brain–Liver Axis: Systemic Inflammatory Integration and Neuroendocrine Consequences

Chronic hepatic inflammation exerts systemic effects extending beyond the gut–liver interface to include neuroendocrine regulation and central stress responses, mediated through bidirectional communication along the gut–brain–liver axis that integrates immune, endocrine, and neural signaling [102,103]. The gut–brain axis comprises neural (e.g., vagal), endocrine, and immune pathways by which gut-derived microbial signals and inflammatory mediators influence hypothalamic and broader central nervous system circuits that regulate appetite, energy balance, and stress responsiveness [103]. Although direct porcine evidence is limited, similar neuroendocrine responses to sustained systemic inflammation have been documented in large mammals, indicating that persistent cytokine signaling can impact feeding behavior, growth performance, and stress physiology via gut–brain–liver communication networks [102].

By dampening systemic inflammatory tone, curcumin may contribute to normalization of these interconnected pathways, with potential implications for animal welfare and productivity. Although direct experimental validation in porcine neurohepatic contexts remains limited, this systems-level perspective provides a coherent mechanistic framework for understanding curcumin’s broader physiological relevance beyond liver-centric endpoints.

#### 6.10.8. Integrative Interpretation: Systems Stabilization Rather than Stage-Specific Therapy

Across the spectrum of NAFLD, NASH, fibrosis, and cirrhosis, curcumin’s biological activity is most coherently interpreted as stabilization of coupled metabolic–inflammatory–oxidative systems rather than as stage-specific therapy, due to its pleiotropic modulation of cellular signaling networks including NF-κB, Nrf2, and TGF-β pathways [104,105]. This systems-level effect reflects the capacity of curcumin to attenuate oxidative stress, suppress pro-inflammatory cytokine production, and inhibit fibrogenic signaling across diverse chronic liver disease models, supporting a unified mechanism of hepatoprotection rather than discrete stage-targeted intervention [104]. This interpretation is particularly appropriate for porcine liver dysfunction, where disease states arise from overlapping metabolic, infectious, and toxic insults, and curcumin’s multi-targeted effects are likely to attenuate interconnected stress responses and pathological loops rather than correct individual pathogenic nodes in isolation [105].

By simultaneously influencing hepatic lipid metabolism, inflammatory signal amplification, oxidative stress, and gut-derived inflammatory inputs, curcumin targets the regulatory architecture that governs disease progression. An integrative summary of curcumin-mediated effects across disease stages and systemic metabolic axes is provided in Table 5.

## 7. Potential Therapeutic Use of Curcumin in the Treatment of Liver Dysfunctions

To place the available mechanistic and experimental findings into a broader translational context, the relevance and limitations of the porcine model relative to other mammalian systems are discussed below.

When considering the potential use of curcumin in the prevention and management of liver diseases in humans, it is necessary to critically evaluate the translational relevance of the porcine model in comparison with other animal systems commonly applied in biomedical research. In vivo pig models represent an important translational intermediary between rodent studies and human clinical investigations, particularly in contexts where anatomical and physiological fidelity is required, such as metabolic, cardiovascular, and surgical research. Nevertheless, important translational limitations remain, including higher experimental costs, limited availability of genetic manipulation tools, and species-specific physiological differences that must be systematically considered [106].

A major advantage of the porcine model is its close anatomical, physiological, and genetic similarity to humans compared with other mammalian species, including sheep, goats, and small rodents, particularly with respect to organ size, metabolic regulation, and immune system architecture. Comparative genomic studies have further demonstrated higher chromosomal synteny and greater similarity of protein-coding regions between pigs and humans than between humans and mice, especially in cancer and metabolic disease models [107]. In contrast, rodent models frequently fail to reproduce complex human disease phenotypes, including those related to cancer progression, xenobiotic metabolism, and immune responses, resulting in limited predictability of toxicity and therapeutic efficacy. These interspecies differences in metabolic rates and xenobiotic receptor systems further constrain direct extrapolation from rodents to humans.

Within this context, porcine models offer a physiologically relevant platform for investigating curcumin-related effects in liver disease, including hepatocellular carcinoma, and may support the development of personalized disease models for precision medicine applications [108]. However, although pigs provide improved translational fidelity compared with rodents, the extensive availability of genetic tools in rodent systems continues to facilitate mechanistic discovery, despite their more limited clinical predictability [109].

Other non-rodent large-animal models, including dogs and non-human primates, also contribute to translational research. Non-human primates share high physiological similarity with humans, particularly in immunological and neurological contexts, but their routine application is constrained by ethical, financial, and logistical limitations. Canine models have been successfully applied in selected hepatic disease contexts, including Wilson’s disease and toxin-induced liver fibrosis models [110,111]; however, breed variability and species-specific physiological differences complicate direct translational interpretation. Ruminant models, such as sheep and goats, although valuable in orthopedic and regenerative research, present major limitations for hepatic metabolism studies due to their distinct digestive physiology and comparatively limited molecular research tools [112].

Overall, current translational research strategies increasingly favor integrated experimental frameworks that combine complementary model systems, using rodents primarily for mechanistic investigations and pigs for physiologically relevant metabolic, toxicological, and nutritional validation. Within this multi-model approach, porcine systems provide a valuable translational bridge between small-animal studies and human clinical research, particularly for evaluating nutritionally derived bioactive compounds such as curcumin.

**Figure 8:**
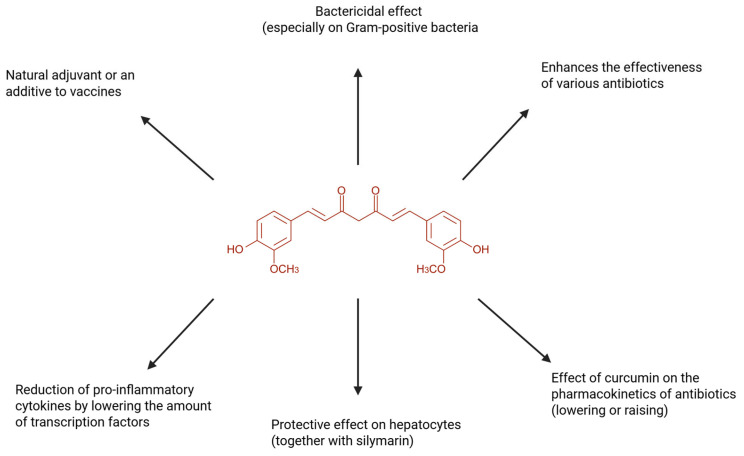
Antibacterial, immunomodulatory and hepatoprotective actions of curcumin and its interactions with antibiotics. Curcumin demonstrates a broad antibacterial activity, particularly against Gram-positive bacteria, and can enhance the effectiveness of several classes of antibiotics by increasing bacterial membrane permeability, disrupting biofilm formation and inhibiting efflux pumps. Figure has been created with www.biorender.com (accessed on 1 May 2025).

When discussing combination strategies involving curcumin, it is important to distinguish between combinations supported by porcine in vivo evidence and those that remain conceptual or extrapolated from non-porcine models. Curcumin has multidirectional antibacterial, anti-inflammatory, immunomodulatory, and hepatoprotective activities that can be leveraged as an adjunct in managing liver dysfunctions in pigs, particularly when hepatic injury is coupled to infection, endotoxemia, or mycotoxin exposure. From a translational perspective, curcumin is best positioned as supportive therapy rather than a standalone treatment, with potential value in three practically relevant scenarios: (i) adjunct to antibiotics in infections associated with hepatic inflammation, (ii) combination with hepatoprotective agents to broaden coverage of oxidative and inflammatory injury mechanisms, and (iii) adjunct to immunotherapies and vaccination strategies to improve the balance between protective antiviral immunity and inflammation-driven collateral tissue damage. Importantly, the potential adjunctive use of curcumin in pigs should be interpreted in a formulation-aware manner, as enhanced-delivery systems cannot be directly equated with conventional curcumin preparations. The proposed interaction points between curcumin, antibiotics, and immune-inflammatory pathways relevant to hepatic protection are summarized in Figure 8.

A comparative overview of curcumin-based combination strategies, indicating the species in which evidence is available and the corresponding evidence status, is provided in Table 6.

From a production perspective, available porcine studies suggest that dietary curcumin supplementation does not induce overt hepatic pathology or acute toxicity under experimental conditions. However, data addressing long-term safety under commercial production settings remain limited. Importantly, systematic evaluation of growth performance, feed efficiency, and residue or depletion kinetics in edible tissues is largely lacking. The absence of such data currently constrains translation to field-level recommendations and highlights the need for longer-duration, production-oriented studies.

Accordingly, while curcumin shows hepatoprotective potential in experimental pig models, the current evidence base is insufficient to support definitive recommendations regarding long-term dietary inclusion rates or production-scale use. Antioxidant effects observed in porcine studies should therefore be viewed as supportive mechanistic signals rather than as standalone indicators of clinically meaningful hepatoprotection. Table 7 summarizes the available human studies on *Curcuma longa* and curcumin, outlining the investigated clinical indications, target conditions or biomarkers, study designs, and key outcomes to enable quick reference.

### 7.1. Adjunct to Antibiotic Therapy: Antibacterial Synergy, Antibiofilm Activity, and Efflux Inhibition

Curcumin can complement antibiotic therapy for infections associated with hepatitis in pigs via two principal routes: direct antibacterial/antibiofilm actions that reduce microbial persistence and pharmacokinetic modulation that increases intracellular antibiotic exposure. In vitro studies indicate that curcumin has bactericidal activity (reported as particularly pronounced against Gram-positive bacteria) and can enhance the effectiveness of diverse antibiotics. For example, combinations of curcumin with antibacterial drugs inhibited growth of *E. coli*, *E. faecalis*, and *S. aureus* more strongly than antibiotics alone, with a synergistic relationship reported in most observations [113]. The most plausible mechanistic explanation is that curcumin weakens bacterial persistence strategies—especially biofilm formation—thereby increasing antibiotic accessibility and susceptibility within structured communities that are otherwise tolerant to treatment. Because biofilms promote chronicity and recurrent inflammation, antibiofilm activity is therapeutically relevant not only for bacterial clearance but also for reducing inflammatory spillover that can exacerbate hepatic injury.

A second mechanism is the effect of curcumin on drug transport. Curcumin has been shown to inhibit efflux transporters (e.g., P-glycoprotein and breast cancer resistance protein (BCRP)) that remove xenobiotics from cells, which can increase intracellular concentrations of antibacterial agents and potentially improve tissue-level efficacy. A concrete example is florfenicol: the addition of curcumin reduced expression and activity of the BCRP transporter in poultry, increasing florfenicol bioavailability and effective exposure [114]. In the context of pig medicine, this is most relevant where intracellular persistence and deep tissue penetration limit antibiotic performance, and where improved intracellular exposure could reduce pathogen burden and, secondarily, inflammation-driven hepatic involvement. On the same logic, tilmicosin (a macrolide used in respiratory infections in pigs) may penetrate tissues and hepatocytes more effectively with curcumin, potentially improving pathogen clearance in systemic inflammatory states that can include hepatic dysfunction. Florfenicol, frequently used in systemic infections in pigs, may similarly benefit from increased effective exposure while curcumin concurrently contributes anti-inflammatory effects in the liver. Overall, curcumin–antibiotic synergy is best summarized as stronger inhibition of microbial growth due to additive antibacterial activity, antibiofilm effects, and increased intracellular antibiotic exposure [113,114].

### 7.2. Combination with Hepatoprotective Agents: Functional Complementarity with Silymarin and UDCA

Curcumin may strengthen outcomes when combined with established hepatoprotective agents used to protect and regenerate damaged liver, particularly silymarin and ursodeoxycholic acid (UDCA). The mechanistic rationale is complementarity: curcumin provides strong anti-inflammatory and endogenous antioxidant-inducing activity, while these agents contribute additional membrane-stabilizing, bile-acid–modulating, and cytoprotective actions.

Silymarin and curcumin protect hepatocytes through partially distinct pathways. Silymarin is known for stabilizing hepatocyte membranes and scavenging free radicals, whereas curcumin more strongly induces endogenous antioxidant systems, including increasing glutathione levels and activity of antioxidant enzymes [115]. In addition, curcumin suppresses inflammatory transcriptional programs (including NF-κB- and AP-1–linked signaling), reducing production of pro-inflammatory cytokines (TNF-α, IL-6) during toxin- or infection-associated injury. In practice, this combination offers broader mechanistic coverage across membrane stability, oxidative injury, and inflammatory amplification, which can translate into stronger protection than either compound alone. Under field-like conditions, a preparation combining curcumin, silymarin, and yeast cell walls (mycotoxin-binding capacity) was evaluated in piglets exposed to fumonisins. The curcumin + silymarin group showed significant improvement in antioxidant status and liver function, including decreased oxidative stress markers (reduced malondialdehyde/TBARS and oxidized proteins) and improved body weight gain relative to controls [116]. This evidence is particularly relevant for pig production settings because chronic mycotoxin exposure is a common driver of subclinical hepatic oxidative stress and reduced performance.

UDCA is standardly used in cholestatic liver damage because it supports bile secretion, exerts anti-apoptotic effects on hepatocytes, and has immunomodulatory effects. Curcumin is a plausible adjuvant to UDCA because it targets inflammatory signaling and oxidative stress in parallel with UDCA’s bile-acid–centric actions. Gheibi et al. reported that combined curcumin + UDCA therapy in a rat non-alcoholic steatohepatitis model protected the liver more effectively than either agent alone, with less hepatocyte apoptosis, lower inflammatory cytokines, and reduced oxidative stress in liver tissue. Combined therapy also improved regeneration and reduced liver enzyme activity in blood compared with monotherapy groups. The authors concluded that synergy results from complementary mechanisms: UDCA improves bile acid metabolism and prevents bile-acid toxicity, while curcumin suppresses inflammation and protects cells from oxidative stress [117]. Although this evidence is not porcine, the mechanistic complementarity supports rational combination strategies in liver dysfunctions where cholestasis and inflammation co-occur.

### 7.3. Adjunct to Immunotherapies and Antiviral Management: Limiting Cytokine-Mediated Collateral Liver Injury

Modern biotechnological approaches in pig health increasingly include immunotherapies, advanced vaccine platforms, RNAi-based interventions, and gene-editing strategies that increase resistance to pathogens. Curcumin, owing to its immunomodulatory and antiviral properties, may function as an adjunct to these approaches by supporting pathogen control while limiting inflammation-driven collateral hepatic injury. In immunotherapy contexts, administration of antiviral cytokines (such as interferon alpha) or stimulation of non-specific immunity can enhance antiviral effectiveness but also risks excessive inflammatory intensity that can damage the liver. Curcumin may provide immune “tone control” by dampening excessive macrophage and lymphocyte activation and reducing cytokine spillover without abolishing protective antiviral activity.

For example, CSFV infection is associated with excessive activation of macrophages and lymphocytes and secretion of high levels of TNF-α and IL-1β, which contribute to tissue injury. Studies in PRRSV-infected pigs have shown that supplementation with curcumin (as a feed additive) reduces key pro-inflammatory cytokines and lowers viral load, suggesting that curcumin can limit harmful inflammatory gain while maintaining effective antiviral defense. Such modulation is valuable in combined immunotherapy because it may improve the benefit–risk balance: sustained antiviral control with less inflammatory liver injury [118].

### 7.4. Curcuminoid-Containing Adjuvants in Vaccination: Improving Immune Response Quality While Constraining Inflammatory Overshoot

Curcumin-related compounds may also have value as vaccine adjuvants or adjuvant additives, potentially increasing immunogenicity while reducing excessive post-vaccination inflammatory signaling. In a study where a combination of *Curcuma zedoaria* extract (containing curcuminoids) and *Astragalus membranaceus* was used as an adjuvant to an inactivated PRRSV vaccine, the additive improved post-vaccination outcomes in piglets—greater body weight gain, higher antibody levels against PRRSV, and stronger proliferation of T and B lymphocytes. Importantly, it also regulated the inflammatory component of the response by inhibiting excessive activation of the TLR4/NLRP3/IL-1β pathway, thereby limiting secretion of pro-inflammatory cytokines (IL-1β, IL-6, TNF-α) after vaccination [119]. This pattern supports a plausible role for curcuminoid-containing adjuvants in improving vaccine response quality by strengthening protective immunity while constraining inflammatory overshoot that can contribute to systemic stress and hepatic burden.

### 7.5. Practical Interpretation: Where Adjunctive Use Is Most Defensible

**Figure 9:**
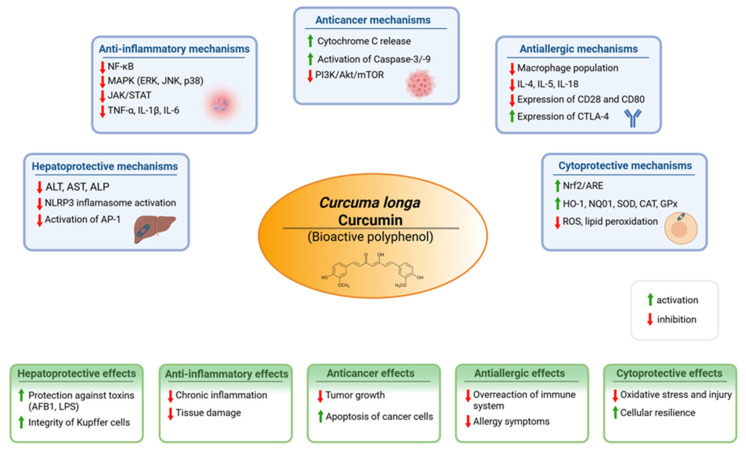
Key mechanisms of action of *Curcuma longa.* The down arrow (↓) indicates a decrease in level, while the up arrow (↑) indicates an increase in the level of a given process.

Overall, the therapeutic value of curcumin in pig liver dysfunction is most convincing when it is positioned as adjunctive support that targets multiple coupled processes—pathogen burden, inflammatory amplification, oxidative stress, and metabolic disruption—rather than as a single-agent treatment. The strongest practical rationale exists for: (i) combination with antibiotics in bacterial infections where biofilm formation and efflux mechanisms contribute to persistence [113,114]; (ii) combination with hepatoprotective agents in toxin- and mycotoxin-associated injury where oxidative stress dominates [115,116]; and (iii) adjunctive use alongside antiviral or vaccine strategies where limiting cytokine-mediated collateral injury is a priority [118,119]. Figure 9 provides an integrative schematic overview of the principal mechanisms of action of *Curcuma longa* (curcumin), highlighting its hepatoprotective, anti-inflammatory, anticancer, antiallergic, and cytoprotective effects, together with the key molecular pathways involved, as supported predominantly by preclinical evidence and selected clinical observations.

## 8. The Effect of Curcumin on the Body

Curcumin (and, in several studies, turmeric/*Curcuma* extracts containing curcuminoids) shows systemic effects that extend beyond the liver and reflect its ability to modulate a small set of conserved biological control nodes. Across tissues and models, the most repeatedly implicated mechanisms include: (i) downregulation of pro-inflammatory transcriptional programs (commonly via NF-κB-linked cytokine and chemokine output), (ii) attenuation of inflammasome-associated signaling (e.g., NLRP3), (iii) activation of cytoprotective stress-response pathways (notably SIRT1- and HO-1–associated responses), and (iv) reinforcement of redox homeostasis (e.g., increased glutathione and reduced lipid peroxidation). Although study designs differ substantially (species, disease models, dose ranges, formulations, and outcome definitions), these mechanistic themes provide a coherent framework for interpreting curcumin-associated effects on inflammation burden, metabolic regulation, barrier function, immune activity, tissue repair, and selected neurobehavioral outcomes.

### 8.1. Inflammation

Curcumin has been reported to reduce inflammatory burden across metabolic, dermatological, musculoskeletal, and gut–immune contexts, typically reflected by decreased pro-inflammatory mediator output (cytokines and eicosanoids) and dampened immune-cell activation.

In obese Wistar rats, Labban’s team observed a significant reduction in body weight (>10%) following treatment with the active compound from *Curcuma longa* versus controls. The same study assessed active substances derived from mangosteen seeds; combined administration produced a stronger reduction in body weight (>14.5%), consistent with additive or synergistic interaction in pathways linking inflammation to metabolic regulation [120].

In parenchymal-cell injury models, an active compound derived from *Curcuma aromatica* protected primary hepatocytes from C57BL/6 mice exposed to paracetamol by inhibiting loss of cell viability and limiting ROS production. The protective effect was associated with activation of the Sirt1/HO-1 signaling pathway, which is mechanistically relevant because SIRT1 influences inflammatory gene expression and stress tolerance, while HO-1 is a key cytoprotective effector induced during oxidative and inflammatory stress [121].

In immune-driven skin conditions, turmeric extract administered as a gel or tonic reduced psoriasis symptoms and was associated with inhibition of potassium channel expression in T lymphocytes and reduced expression of pro-inflammatory cytokines (including IL-17A, IL-17F, and IL-22). The same preparation reduced symptoms of eczema and atopic dermatitis, consistent with down-modulation of inflammatory T-cell–linked pathways in the skin [122].

Curcumin also modulates eicosanoid-related inflammatory signaling. It inhibited leukotriene formation in rat peritoneal polymorphonuclear neutrophils and inhibited TNF-α–dependent NF-κB activation, aligning with reduced upstream inflammatory amplification. In a rat tendon injury model, curcumin administration promoted deposition of well-organized collagen fibers—an outcome consistent with improved repair quality in tendinopathy, where excessive inflammation commonly disrupts structured extracellular matrix remodeling [123].

Beyond classical inflammation markers, curcumin has been linked to cytoprotective signaling that intersects with inflammatory injury. It stimulated phosphorylation of mTOR kinase and increased SIRT1 expression and protected kidneys against gentamicin-induced damage by reducing apoptosis of tubular cells. In parallel, it decreased foam cell formation and intracellular lipid accumulation, linking inflammatory regulation to lipid-handling phenotypes relevant to chronic inflammatory disease processes [124].

Curcumin has also been associated with clinically relevant inflammation–metabolism endpoints. In humans with metabolic syndrome, curcumin increased the heavy HDL fraction while decreasing the light LDL fraction and triglycerides. It also affected leptin signaling by interrupting this pathway and activated PPAR-γ in rat hepatic stellate cells—an observation relevant to inflammation–fibrosis coupling because stellate-cell activation is sensitive to inflammatory cues and governs remodeling programs [125]. In patients with knee osteoarthritis, twice-daily supplementation for four weeks using capsules containing turmeric, ginger, and black pepper reduced expression of prostaglandin PGE2, consistent with reduced eicosanoid-mediated inflammatory activity [126].

Finally, curcumin has been reported to act at the gut–barrier–immune interface. Rather than preventing “LPS production,” the more defensible interpretation is that curcumin attenuates LPS-driven downstream consequences (barrier disruption and cytokine induction), in part through modulation of intestinal alkaline phosphatase (iALP), an enzyme linked to luminal detoxification processes. Curcumin also reshaped gut microbiota composition, with reported shifts involving Prevotellaceae, Bacteroidaceae, and Rikenellaceae and increases in butyrate-producing taxa (*Clostridium* cluster IV and subclasses of *Clostridium*), a pattern biologically consistent with barrier-supportive metabolic output and reduced systemic inflammatory pressure [127].

### 8.2. Oxidative Stress

Curcumin demonstrates antioxidant effects in multiple tissues, generally expressed as reduced oxidative burden together with strengthening of endogenous redox-buffering capacity—changes that can secondarily reduce inflammation amplification.

Administration of curcumin to rats increased total glutathione and reduced hepatic ROS accumulation; reduced nitrogen oxides were also observed, consistent with attenuation of both oxidative and nitrosative stress [121]. In IUGR-related oxidative injury, curcumin significantly lowered MDA and hydrogen peroxide, supporting reduced lipid peroxidation and peroxide load under systemic stress conditions [128].

Formulation and route appear to influence tissue targeting in oxidative-stress contexts. Intranasal administration of curcumin nanomicelles was reported to influence the ocular surface system via delivery to the ophthalmic branch of the trigeminal ganglion, restoring homeostasis through reduced ROS production and pro-inflammatory mediator expression alongside increased neurotrophic factors. In mice, conjunctival curcumin administration reduced dry eye syndrome symptoms and decreased secretion of IL-4 and IL-5, linking redox modulation to reduced local cytokine output in a mucosal surface environment [128].

Cardiac models further support a cytoprotective role in oxidative injury. Curcumin reduced cardiomyocyte hypertrophy and TNF-α–induced cardiotoxicity and attenuated toxicity induced by peptidoglycan exposure or hypoxia/reoxygenation mechanisms in rat cardiomyocytes. It reduced ischemia/reperfusion toxicity via activation of the SIRT1 pathway and attenuated oxidative-stress effects linked to NADPH and PI3K/Akt signaling. In primary human ventricular cardiomyocytes (HVCM), curcumin reduced necrosis and ROS production, restored mitochondrial membrane potential, and improved glucose transporter-related parameters, collectively consistent with preservation of mitochondrial function and metabolic stability during oxidative stress [129].

### 8.3. Obesity

Curcumin has been proposed as supportive in obesity management primarily through attenuation of chronic low-grade inflammation and downstream metabolic correction. Curcuminoids promoted browning of adipose tissue by inhibiting chronic inflammation in white adipose tissue adipocytes through modulation of TNF-α signaling, consistent with a shift toward a more metabolically active adipose phenotype [129]. Improvements in lipid profiles have also been reported, including increased HDL and ApoA and decreased LDL, ApoB, and the ApoB/ApoA ratio, indicating a less atherogenic lipoprotein pattern accompanying anti-inflammatory metabolic improvement [130].

### 8.4. Immune System

Curcumin modulates innate and adaptive immune functions, with reported effects on macrophage activity, inflammasome signaling, cytokine output, costimulatory pathways, and selected antiviral-relevant mechanisms. In vitro studies demonstrated that curcumin reduces macrophage population metrics, inhibits NLRP3 inflammasome signaling, and suppresses downstream NF-κB-linked expression of TNF-α, IL-6, IL-1β, and IL-18 [131]. These effects are mechanistically aligned with reduced innate-inflammatory amplification and may be relevant to systemic inflammatory load that secondarily affects organ function, including the liver.

Curcumin has also been described to exert immunosuppressive or immune-balancing effects depending on activation context. It reduced expression of costimulatory molecules CD28 and CD80, increased CTLA-4 expression on macrophages, and increased IL-10 levels. In human macrophages exposed to cigarette smoke, IL-8 decreased by 85% via NF-κB suppression, illustrating marked suppression of high-intensity inflammatory output in activated innate immune cells. In gastrointestinal inflammation, oral curcumin combined with resveratrol alleviated acute enteritis by down-regulating Th1 responses and preventing bacterial translocation via maintenance of intestinal barrier function. Importantly for pig physiology, curcumin and resveratrol modulated gut microbiota of weaned piglets, down-regulated the TLR4 signaling pathway, alleviated intestinal inflammation, and enhanced intestinal immune function, indicating immune modulation in a pig-relevant setting with plausible systemic implications [132].

Multiple antiviral-associated effects have been reported and should be interpreted as mechanistic or preclinical observations rather than established therapeutic claims. Curcumin has been discussed in SARS-CoV-2-related contexts based on broader antiviral activity patterns, including suspected activity against HIV-related targets involving concepts of protease and integrase inhibition. Zika and chikungunya viruses were reported to lose infectivity in association with changes in angiotensin receptor expression (reduced AT1 and increased AT2) following curcumin supplementation [131]. In hepatocyte infection biology, curcumin decreased HBV surface antigen (HBsAg) production and reduced cccDNA copies in nuclei of infected hepatocytes, inhibiting HBV replication; this was accompanied by reduced acetylation of histones H3 and H4 associated with cccDNA. Curcumin also inhibited transcription of the HBV X gene (HBx) via a p53-dependent pathway without cytotoxicity to liver cells. Additionally, curcumin disrupted AP-1 binding activity in HeLa cells, reducing transcription of HPV-18 genes, consistent with suppression of transcription factor–dependent viral gene expression [133].

### 8.5. Improving Cognitive Function

Curcumin has been linked to neurocognitive and neurobehavioral outcomes through two principal lines of evidence: gut microbiome modulation (gut–brain axis) and direct effects on neurochemical signaling and stress-response pathways.

Extract from *Curcuma longa* rhizome influenced the human gut microbiome, increasing abundance of bacteria from Bacteroidaceae, Desulfovibrionaceae, Rikenellaceae, and Lachnospiraceae, and at the genus level *Clostridium* spp., *Bacteroides* spp., *Blautia*, and *Enterobacter* spp. An increase in propionate- and butyrate-producing taxa was observed, while *Citrobacter freundii*, *Enterococcus faecalis*, *Shigella dysenteriae*, and *Escherichia coli* decreased. In male Wistar rats receiving an alcohol extract of turmeric containing curcumin and a turmeric oil fraction, longitudinal shifts were reported: after three months, total aerobic bacteria and *Lactobacillus* decreased, while total anaerobic bacteria, *Clostridium perfringens*, and coliform bacteria increased; after longer treatment (two years), the latter decreased substantially, suggesting time-dependent remodeling rather than a single-direction shift [134].

Curcumin may also support post-exercise recovery. In exercised mice, reductions in circulating markers of muscle damage and inflammation were observed, and curcumin reduced muscle soreness, consistent with attenuation of exercise-induced inflammatory signaling [135].

In mood and neurological-condition contexts, curcumin reduced monoamine degradation in mice via MAO-A and MAO-B mechanisms, increasing serotonin and dopamine levels in brain tissue. It may mitigate stress-related effects by restoring hippocampal signaling involving BDNF and CREB. Reported outcomes include reduced severity, duration, and frequency of migraine attacks, reduced motor disturbances in Parkinson’s disease, and improved clinical condition in patients with amyotrophic lateral sclerosis [136].

### 8.6. Lifestyle Diseases

Curcumin has been reported to influence metabolic control and vascular/tissue remodeling processes relevant to lifestyle-associated disease phenotypes. It induced glucose uptake and activity of glucose transporter 2 (GLUT2) and supported insulin production, consistent with improved glucose handling [137].

In ischemia-related models, curcumin showed anti-fibrotic and pro-repair effects. In mice with hindlimb ischemia induced by double ligation of the femoral, saphenous, and circumflex iliac arteries, intravenous curcumin reduced skeletal muscle fibrosis and increased muscle fiber density. In a related model involving femoral artery ligation and excision, two weeks of curcumin improved perfusion recovery and increased capillary density, consistent with pro-angiogenic effects that support functional tissue recovery after ischemic injury [138]. Table 8 summarizes the clinical applications of *Curcuma longa* and its main component, curcumin.

## 9. Drug–Nutrient Interactions and Veterinary Drug Compatibility

Curcumin is frequently positioned as an adjunctive compound in veterinary settings because it can influence inflammation, oxidative injury, and microbial persistence. However, it should not be treated as a biologically inert feed supplement during pharmacotherapy. Curcumin (and curcuminoid-containing preparations) can plausibly modulate host drug-handling systems, creating interaction potential with veterinary medicines. In pigs, this consideration is amplified by (i) frequent polypharmacy during infectious and inflammatory episodes, (ii) reliance on oral administration and variable feed intake during illness, and (iii) the practical constraints of food-animal medicine, where altered exposure may affect both safety and residue depletion kinetics. Interaction magnitude is also formulation-dependent: enhanced-bioavailability systems and co-administered bioenhancers may increase systemic exposure and therefore increase the likelihood of clinically relevant interactions [139].

### 9.1. Transporter-Level Interactions: Implications of P-gp and BCRP Modulation

Barrier and excretory tissues express efflux transporters that regulate absorption and elimination by exporting xenobiotics out of cells. In the intestine, transporters such as P-glycoprotein (P-gp/ABCB1) and breast cancer resistance protein (BCRP/ABCG2) can limit oral bioavailability by returning substrates to the intestinal lumen. In the liver and biliary tract, transporter activity contributes to canalicular efflux and biliary elimination. Curcumin has been reported to inhibit transporter function and/or reduce transporter expression in experimental systems, which can increase intracellular and tissue exposure to transporter-substrate drugs [140,141,142].

From a veterinary perspective, this mechanism has dual consequences. On one hand, increased intracellular exposure may support efficacy for medicines whose activity depends on tissue penetration or intracellular availability. On the other hand, higher exposure can increase the probability of concentration-dependent adverse effects and may shift residue depletion dynamics in food animals. Therefore, transporter modulation should be presented as a benefit–risk determinant rather than as an unqualified advantage.

### 9.2. Metabolic Interactions: Phase I/Phase II Modulation and Disease-State Amplification

Curcumin can interact conceptually with hepatic metabolism through modulation of phase I oxidation and phase II conjugation systems. The practical interpretation is straightforward: inhibition of metabolic pathways can increase systemic exposure and prolong half-life, whereas induction can reduce exposure and compromise efficacy. Disease state is a key amplifier. When curcumin is administered in the very settings where it is most often proposed—ongoing hepatic injury, systemic inflammation, endotoxemia, or mycotoxin exposure—baseline clearance may already be reduced. In these contexts, even moderate modulation of metabolic capacity or biliary transport can disproportionately alter drug exposure compared with healthy animals.

### 9.3. Compatibility with Antimicrobials: Distinguishing Synergy from Exposure Modification

Curcumin is frequently discussed as an adjunct to antimicrobial therapy because it can affect bacterial persistence mechanisms (notably biofilm formation) and may increase effective antibiotic exposure at the site of action. In a review context, it is important to distinguish two non-identical concepts: (i) true pharmacodynamic synergy at the microbial target (e.g., combination effects that exceed additivity) and (ii) host-mediated exposure modification (e.g., altered absorption, distribution, or elimination that changes antibiotic concentrations). Both can improve apparent efficacy, but they have different implications for safety, dosing, and residue management.

For interpretability, studies claiming curcumin–antibiotic synergy should, at minimum, use standardized combination endpoints (e.g., recognized MIC-based combination metrics and bactericidal time-kill approaches) and, for antibiofilm claims, include quantitative biofilm endpoints that reflect both biomass and viable bacterial burden. Where intracellular persistence is part of the rationale, intracellular killing or intracellular accumulation measures materially strengthen translational plausibility. When interaction mechanisms are attributed to transporters or metabolism, the supporting evidence should include at least one direct readout (expression/activity proxy) rather than relying on efficacy outcomes alone.

### 9.4. Practical Risk Management in Swine Medicine and Production Settings

In pigs, compatibility considerations extend beyond immediate clinical safety to include regulatory and production constraints. Curcumin should be treated as a pharmacologically active co-factor during drug treatment courses, particularly when administered in formulations designed to increase systemic exposure. Increased caution is most appropriate during: (i) polypharmacy, (ii) long-acting drug formulations where altered clearance could extend exposure windows, and (iii) treatment of animals with marked systemic inflammation or hepatic dysfunction where baseline clearance is reduced.

From a translational perspective, studies combining curcumin with veterinary medicines should report formulation, dose (including feed concentration where relevant), timing relative to drug administration, duration of co-administration, and monitoring for signals consistent with altered exposure (changes in efficacy, adverse effects, or pharmacokinetic indicators where available). In food-animal contexts, the lack of residue depletion data under curcumin co-administration should be stated explicitly as a limitation when relevant. To systematically delineate the relationships between traditional applications of *Curcuma longa* and the available mechanistic and experimental evidence, a summary table (Table 9) was constructed, highlighting reported biological effects, identified knowledge gaps, and the level of translational support across different experimental models.

## 10. Biomarkers and Endpoints for Evaluating Curcumin Effects in Porcine Liver Dysfunction

A persistent weakness across the curcumin literature is heterogeneity in endpoints and reporting standards, which limits cross-study comparability and makes mechanistic conclusions difficult to validate. In porcine liver dysfunction, this limitation is particularly important because many models involve mixed injury mechanisms (oxidative stress, inflammation, cholestasis, metabolic disruption, and variable regeneration). A structured endpoint framework improves interpretability by linking claimed mechanisms to measurable outcomes and by separating biochemical improvement from true structural preservation.

### 10.1. Recommended Endpoint Framework (Core and Mechanistic Tiers)

To support interpretable hepatoprotection claims in pigs, endpoints should be organized into a core clinical chemistry tier, a mechanistic tier (oxidative and inflammatory readouts), and mandatory histology standards. A recommended minimum dataset and associated confirmatory measures are summarized in Table 10.

### 10.2. Sampling Strategy, Time Structure, and Pre-Analytical Control

Because porcine liver injury models often show time-dependent injury and recovery, single time-point sampling can misrepresent effects. Where feasible, studies should align sampling to the biology of the model (baseline, peak injury, and early recovery) rather than relying on endpoint-only comparisons. Pre-analytical handling is particularly important for oxidative endpoints: liver tissue should be processed rapidly and stored appropriately to reduce artifactual oxidation. Reporting should specify liver region sampled, handling conditions, assay type, and normalization strategy.

### 10.3. Histology Requirements for Strong Hepatoprotection Claims

Histology should be treated as the decisive evidence layer, particularly when conclusions extend beyond “biochemical improvement” to claims of protection, regeneration, or antifibrotic activity. Minimum expectations include standardized scoring on H&E and blinding of the assessor. For steatosis-driven phenotypes, standardized grading should distinguish relevant patterns where possible. For chronic injury models, fibrosis staining with semi-quantitative scoring is expected, and antifibrotic claims should be supported by collagen-related measures and/or stellate activation markers rather than inferred from enzymes alone.

### 10.4. Model-Sensitive Endpoint Alignment

Endpoint emphasis should be matched to the dominant injury mechanism. In acute toxin/endotoxin models, the most informative dataset combines core enzymes with oxidative stress indices, cytokines, pathway-level readouts, and H&E-based injury scoring. In mycotoxin exposure models, inclusion of bile acids and longer-term oxidative and histological measures improves interpretability, while performance outcomes may support translational relevance. In infection-associated hepatitis, immune activation measures and pathogen burden proxies strengthen causal inference, particularly when antibiotics are co-administered. In chronic injury or fibrosis-oriented models, fibrosis staining and stellate activation measures should be treated as mandatory endpoints, and repeated time points are strongly preferable to distinguish prevention of progression from true regression.

## 11. Human Nutrition

Human health, particularly in the context of the increasing prevalence of lifestyle-related non-communicable diseases, has become a major focus of contemporary scientific research. A substantial proportion of these conditions is closely associated with unfavorable lifestyle factors, including chronic psychological stress and inappropriate dietary patterns characterized by a high intake of ultra-processed foods. These factors are widely recognized as important determinants of metabolic disturbances, low-grade chronic inflammation, and oxidative stress, which play a key role in the pathogenesis of many civilization-related diseases.

In this context, growing attention has been directed toward dietary bioactive compounds which, in combination with broader lifestyle modifications, may contribute to the maintenance of human health. One such compound is curcumin, a natural polyphenolic substance derived from *Curcuma longa*. Curcumin has a long history of use in traditional medical systems, including Ayurvedic medicine, where turmeric-based preparations have been employed for centuries. Contemporary research in nutrition and biomedical sciences is currently focused on elucidating the biological mechanisms of curcumin and evaluating its potential relevance to human health within modern dietary patterns.

Curcumin is widely used as a culinary spice in South and Southeast Asian cuisines. From a human nutrition perspective, it represents a dietary bioactive component, with intake occurring primarily through the regular use of turmeric as a culinary ingredient rather than in the form of concentrated pharmaceutical preparations [49,143]. Estimated daily dietary intake of curcumin is strongly dependent on dietary habits and cultural background. In populations where turmeric is routinely used as a spice, average curcumin intake is estimated at approximately 60–100 mg per day, whereas in Western diets its consumption is negligible or virtually absent [144]. In contrast to supplementation, dietary exposure to curcumin is characterized by low doses consumed chronically, which is particularly relevant from a nutritional standpoint. Such long-term, low-dose intake reflects physiological dietary conditions and may result in subtle but biologically meaningful modulation of inflammatory and oxidative processes, without the risks associated with high pharmacological doses [49,145].

Despite the documented biological effects of curcumin, its potential health benefits are substantially limited by poor oral bioavailability. This limitation is primarily attributed to inadequate intestinal absorption, rapid metabolic transformation, and fast systemic elimination following oral administration. To date, several studies have investigated the pharmacokinetic profile of curcumin in humans, consistently demonstrating very low plasma concentrations after ingestion of native curcumin. The highest plasma concentration reported in humans was 0.051 µg/mL following oral administration of 12 g of curcumin [60].

A comprehensive investigation of curcumin oral bioavailability in healthy adults was conducted by Schiborr et al., who compared native curcumin powder, micronized curcumin, and liquid micellar formulations. In this randomized crossover study, healthy volunteers (13 women and 10 men) received a single oral dose of 500 mg curcuminoids administered as native powder, micronized powder, or liquid micelles composed of 7% curcumin powder (corresponding to 6% curcumin) and 93% Tween-80. Blood samples were collected over a 24 h period for pharmacokinetic analysis [146].

The results demonstrated that administration of curcuminoids in micellar form (410 mg curcumin) resulted in a markedly higher mean peak plasma concentration (C_max = 3228 nmol/L) compared with native curcuminoids, for which the C_max reached only 7 nmol/L. Moreover, peak plasma concentrations achieved after ingestion of 410 mg micellar curcumin (3.7 µmol/L in women and 2.6 µmol/L in men) exceeded those previously reported following the intake of 8 g of native curcumin (1770 nmol/L) [147].

Turmeric and curcumin are considered safe for human consumption, particularly when administered orally. They have also been shown to be safe in animal studies, demonstrating no mutagenic effects and no adverse outcomes during pregnancy. Nevertheless, further studies in humans are recommended. Oral administration of curcumin at doses up to 6 g/day for periods of 4–7 weeks has been reported to be safe, although minor gastrointestinal disturbances may occur [50].

Among the three principal curcuminoids, all exhibit biological activity with potential therapeutic relevance; however, curcumin has become the primary focus of research interest. This is largely due to the growing body of evidence suggesting its potential beneficial effects in humans, which has led to research efforts being concentrated predominantly on this compound [148]. This trend is supported by a recently published systematic review and meta-analysis of randomized controlled trials, evaluated using the GRADE (Grading of Recommendations Assessment, Development, and Evaluation) methodology, which demonstrated that curcumin supplementation may contribute to reductions in inflammation and oxidative stress in adults aged ≥ 18 years across a range of health conditions [149].

Over recent decades, the therapeutic potential of curcumin has become increasingly apparent across multiple areas of biomedical research. Numerous experimental and preclinical studies indicate its activity against a broad spectrum of cancers, including chemoresistant colorectal cancer, as well as esophageal, thyroid, and skin cancers [150]. In parallel, curcumin has been shown to possess pronounced anti-inflammatory properties, which represent one of the key mechanisms underlying its biological activity [151].

The anticancer mechanisms of curcumin are multifaceted and involve modulation of numerous intracellular signaling pathways, including growth factors, protein kinases, inflammatory mediators, and transcription factors. These interactions lead to inhibition of tumor cell proliferation, reduction in invasive capacity, and suppression of metastatic potential [150].

Beyond oncology, curcumin has also been investigated in the context of various other conditions, including respiratory tract infections, non-alcoholic fatty liver disease, skin photoaging, Parkinson’s disease, obesity, diabetes, HIV-associated diarrhea, and Alzheimer’s disease. In neurodegenerative disorders, its effects have been attributed in part to inhibition of β-amyloid aggregation and oligomer formation [104,152].

At the molecular level, curcumin is capable of interacting with multiple key components of oncogenic signaling pathways, particularly protein kinases. It has been shown to inhibit protein kinase C through stable interactions with amino acid residues within the C1B subdomain, as well as to effectively bind to the active site of glycogen synthase kinase-3β. Additionally, curcumin has been reported to inhibit phosphorylase kinase via a non-competitive mechanism. Cyclin-dependent kinases, including CDK1, CDK2, and CDK4/6, play a fundamental role in cell cycle regulation, and their dysregulation is a critical process in cancer development; curcumin has demonstrated the ability to modulate the activity of these enzymes, further highlighting its multi-target mode of action [153].

In summary, curcumin should be considered primarily as a dietary bioactive compound whose potential relevance to human health arises from regular, long-term consumption within habitual dietary patterns rather than from short-term, high-dose interventions. Its presence in the diet, particularly in combination with other plant-derived components, aligns with contemporary nutritional approaches based on holistic dietary patterns and the concept of functional foods.

From a human nutrition perspective, it is also important to note that curcumin exhibits a favorable safety profile when consumed as part of the diet, as supported by its long-standing culinary use and available toxicological data. Accordingly, curcumin may represent a valuable component of a health-supporting diet, provided that its role is interpreted within the context of overall dietary patterns rather than as a substitute for pharmacological treatment.

## 12. Summary

The liver plays a key role in metabolism and maintaining homeostasis in animals, including pigs, as it is responsible for glucose, lipid and protein metabolism and detoxification. Liver inflammation in pigs is often the result of infections, toxins or unfavorable environmental conditions, and its symptoms are usually clinically invisible, detected mainly histopathologically. Curcumin, the active ingredient of turmeric, has a wide range of health-promoting properties, including anti-inflammatory, antioxidant and hepatoprotective properties, which makes it a potential agent in the treatment of liver inflammation in pigs. Curcumin acts at the molecular level by modulating signaling pathways such as NF-κB, MAPK kinases and the JAK/STAT pathway, which leads to a decrease in the secretion of pro-inflammatory cytokines (e.g., TNF-α, IL-6) and an increase in the level of anti-inflammatory cytokines (e.g., IL-10). It also acts as a strong antioxidant, activating the Nrf2 pathway, which helps protect tissues from oxidative stress. In addition, curcumin affects immune cells, reducing the activation of pro-inflammatory pathways and regulating the cytokine response. Curcumin also has a protective effect on hepatocytes, reducing lipid peroxidation, lowering the activity of cytolytic enzymes and regulating the expression of genes related to apoptosis. It inhibits the replication of viruses such as CSFV and PRRSV by affecting lipid metabolism and cytokine response. Due to its antiparasitic properties, curcumin can also play a protective role in liver diseases of various aetiologies. In combination with antibiotic therapy, curcumin can support the treatment of hepatitis in pigs. Its immunomodulatory, antiviral, and hepatoprotective effects indicate its great potential in the treatment of liver diseases, both in prevention and therapy. In recent years, advanced delivery technologies have significantly enhanced the therapeutic potential of curcumin in veterinary medicine. Novel nanoformulations—such as polymeric nanoparticles, solid lipid nanoparticles, liposomes, and curcumin–phospholipid complexes—allow for a several-fold increase in its bioavailability and controlled release, enabling more efficient targeting of hepatic tissue. Experimental studies demonstrate that nanocurcumin accumulates more effectively in inflamed liver regions, where it modulates local immune responses with far greater potency than conventional formulations. Moreover, high-throughput omics methods (transcriptomics, proteomics, metabolomics) have revealed that curcumin reshapes entire metabolic networks in hepatocytes, including mitochondrial bioenergetics, lipidomic remodeling, and epigenetic regulation via histone acetylation and microRNA expression. These findings suggest that curcumin may act not only as a symptomatic anti-inflammatory agent but also as a regulator of long-term hepatic reprogramming. Emerging precision-livestock technologies, such as non-invasive biosensor monitoring of liver stress markers (e.g., ROS signatures, inflammatory cytokines, or metabolic fluxes), open possibilities for integrating curcumin-based preventive strategies with real-time detection of early hepatic dysfunction in pigs. Combined with advances in AI-driven prediction models for hepatotoxicity and nutrient-metabolism interactions, curcumin is increasingly recognized as a promising component of next-generation, personalized nutritional and therapeutic interventions in swine health management.

Taken together, the accumulated evidence indicates that curcumin should not be regarded solely as a single-target anti-inflammatory compound, but rather as a multifunctional modulator of hepatic homeostasis operating across inflammatory, oxidative, metabolic, and immunological axes. This broad mode of action may be particularly advantageous in complex, multifactorial liver disorders in pigs, where subclinical inflammation, metabolic dysregulation, and environmental stressors frequently coexist. Nevertheless, the translation of these promising findings into routine veterinary or nutritional practice requires further refinement. Critical challenges remain with respect to the standardization of curcumin formulations, dosing regimens, and treatment duration, as well as the identification of robust, clinically meaningful liver-specific endpoints in swine. In this context, future research should prioritize controlled, pig-specific intervention studies that integrate conventional pathological assessment with emerging precision-monitoring tools, thereby enabling early detection of hepatic dysfunction and objective evaluation of intervention efficacy. Incorporating curcumin-based strategies into evidence-driven livestock management frameworks may ultimately contribute to improved liver resilience, reduced disease burden, and more sustainable production systems. However, defining its optimal role will depend on continued interdisciplinary efforts linking nutritional science, veterinary medicine, formulation technology, and systems biology.

## Figures and Tables

**Table 1:** Qualitative indicators applied to assess internal validity, robustness, and translational relevance of included studies and to guide narrative evidence weighting.

Quality Appraisal Domain	Operational Criterion Used in This Review
**Curcumin identity and formulation**	Clear specification of curcumin/curcuminoid identity, source, purity, and formulation (e.g., conventional extract, nano formulation, phospholipid complex, bioenhancer-containing preparation)
**Dose and** **administration**	Transparent reporting of administered dose (mg/kg), route (oral/feed-based vs. other), dosing frequency, and duration
**Experimental** **controls**	Use of appropriate negative and/or positive control groups enabling attribution of observed effects to curcumin intervention
**Model relevance**	Relevance of the experimental model to porcine liver dysfunction or hepatitis (toxin-, endotoxin-, infection-, or metabolism-associated injury)
**Hepatic outcome** **relevance**	Inclusion of functionally meaningful hepatic endpoints (e.g., ALT, AST, LDH, lipid accumulation, bilirubin)
**Histopathological assessment**	Use of liver histology to corroborate biochemical or molecular findings where hepatoprotection is claimed
**Oxidative stress and inflammation**	Reporting of oxidative stress markers and/or inflammatory mediators measured in liver tissue or circulation
**Exposure** **plausibility**	Coherence between dose, formulation, route of administration, and plausibility of achieving biologically relevant exposure in pigs
**Methodological transparency**	Sufficient methodological detail to permit interpretation, comparison across studies, and identification of limitations
**Consistency across endpoints**	Concordance between biochemical, molecular, and structural outcomes supporting internal consistency of conclusions

**Table 2:** Summary of major infectious and toxic etiological factors contributing to liver dysfunction in pigs and their convergent cellular and molecular mechanisms. Despite distinct initiating insults, viral, bacterial, and toxin-mediated hepatic injuries converge on shared intrahepatic pathways, including Kupffer-cell activation, cytokine amplification, oxidative stress, hepatocyte death (apoptosis/necrosis), and fibrogenic remodeling [13,14,15,16,17,18,19,20,21,22,23,24,25].

Etiological Factor	Primary Hepatic Targets	Hallmark Pathological Features	Dominant Cellular Mediators	Key Molecular/Signaling Pathways	Representative Outcomes
**Hepatitis E virus (HEV)**	Hepatocytes	Immune-mediated lobular inflammation; usually subclinical; minimal macroscopic changes	Lymphocytes, Kupffer cells	Cytokine-mediated inflammation; immune activation	Asymptomatic hepatic inflammation; zoonotic transmission risk [13]
**Porcine circovirus type 2 (PCV2)**	Hepatocytes, macrophages	Lobular inflammation; apoptotic necrosis (apoptotic bodies); architectural disorganization; periportal/perilobular fibrosis in chronic cases	Kupffer cells, hepatocytes, T lymphocytes	Caspase-mediated apoptosis; pro-inflammatory cytokines; fibrogenic signaling	Chronic hepatitis; parenchymal jaundice in severe cases; PCVAD-associated liver injury [16,17,19]
***Salmonella enterica* (septicemia)**	Hepatocytes, sinusoidal macrophages	Scattered necrotic foci (paratyphoid nodules); microabscesses; granulomas (rare chronic cases)	Kupffer cells, neutrophils, macrophages	Cytokine release; oxidative stress; innate immune activation	Acute systemic disease with hepatic involvement; limited chronic liver dysfunction after recovery [20]
***Leptospira* spp.**	Hepatocytes, sinusoidal endothelium	Diffuse or focal hepatocyte necrosis; endothelial damage; cholestasis; parenchymal jaundice	Kupffer cells, endothelial cells	Toxic-enzymatic injury; immune-mediated inflammation	Elevated ALT/ALP; high neonatal mortality; reproductive losses in sows [21]
**Aflatoxins (AFB_1_)**	Hepatocytes (centrilobular zones)	Centrilobular degeneration and necrosis; steatosis; fibrosis; cirrhosis with prolonged exposure	Hepatocytes, Kupffer cells	CYP-mediated bioactivation; DNA/protein adduct formation; oxidative stress	Reduced growth performance; immunosuppression; hepatocellular carcinoma (rarely detected) [23]

**Table 3:** Characteristics of porcine in vivo studies assessing curcumin supplementation, including dose, formulation, duration, hepatic endpoints, and exposure-related information.

Porcine Model/Context	Dose (mg/kg)	Formulation	Duration	Key Hepatic Endpoints Assessed	Reported Hepatic Effects	Exposure/PK Data Reported
**Intrauterine growth** **restriction-associated** **oxidative stress**	~200 mg/kg	Conventional curcumin	Several weeks	MDA, protein carbonyls, SOD, CAT, Nrf2 target genes	Reduced lipid peroxidation; increased antioxidant enzyme activity; induction of HO-1, GST, NQO1	No
**Endotoxin (LPS)-induced hepatic injury**	300–400 mg/kg	Feed-supplemented curcumin	Acute to short-term	AST, LDH, hepatic lipids, apoptosis markers	Lower AST/LDH; reduced hepatic lipid accumulation; modulation of apoptosis-related genes	No
**Viral infection models (CSFV, PRRSV)**	Variable (in vitro–in vivo combined evidence)	Conventional curcumin	Short-term	Viral replication, cytokines, liver injury markers	Inhibition of viral replication (primarily in vitro); reduced inflammatory burden in vivo	No
**Mycotoxin-associated oxidative stress (combination strategies)**	Variable (as part of mixed supplements)	Curcumin-containing combinations	Weeks	TBARS/MDA, antioxidant status, growth indicators	Improved antioxidant status; partial mitigation of toxin-associated liver stress	No
**Safety/chronic supplementation studies**	Up to several hundred mg/kg	Conventional curcumin	Weeks to months	Histology, inflammatory infiltrates	No overt liver pathology; no inflammatory damage reported	No

**Table 4:** Comparative overview of porcine and non-porcine evidence supporting curcumin-mediated hepatoprotective mechanisms across key outcome domains. The table explicitly distinguishes direct porcine in vivo support from mechanistic inference based on non-porcine liver models.

Outcome Domain	Porcine In Vivo Evidence	Porcine Liver Tissue/Cell Evidence	Non-Porcine Liver Models	Strength of Porcine Support	Notes on Translational Interpretation
**Liver enzymes (ALT, AST, LDH, ALP)**	Yes—reduced leakage enzymes in piglets exposed to endotoxin or oxidative stress models	Limited	Yes (rodents, humans)	Direct	Concordant reductions in enzymes indicate hepatocellular membrane stabilization; functional relevance strongest when combined with histology
**Histopathology** **(necrosis, steatosis,** **lipid accumulation)**	Partial—reduced lipid accumulation and structural injury in endotoxin-associated models	No	Yes (toxin-, diet-, and ischemia-induced models)	Indirect	Histological preservation in pigs is model-dependent; strongest support in endotoxin-related injury
**Oxidative stress** **markers (MDA, ROS, GSH, SOD, CAT)**	Yes—consistent reduction in lipid peroxidation and enhancement of antioxidant defenses	Yes (gene expression)	Yes	Direct	Redox modulation is the most robust and reproducible porcine-supported mechanism
**Nrf2/ARE pathway** **activation**	Yes—increased expression of HO-1, GST, NQO1 in porcine tissues	Yes	Yes	Direct	Provides mechanistic basis for antioxidant and cytoprotective effects in pigs
**NF-κB–related** **inflammatory signaling**	Limited (cytokine outputs only)	No direct NF-κB readouts	Yes (extensive)	Extrapolated	In pigs, reduced cytokines imply upstream modulation, but direct pathway validation is lacking
**NLRP3 inflammasome activity**	No	No	Yes	Extrapolated	Presented as conserved mechanism inferred from macrophage- and rodent liver studies
**Pro-inflammatory cytokines (TNF-α, IL-1β, IL-6)**	Yes—reduced circulating and hepatic cytokines	Limited	Yes	Indirect	Cytokine suppression reflects anti-inflammatory tone but does not alone prove direct antiviral action
**Viral infection–associated liver injury (CSFV, PRRSV)**	Yes—reduced viral load and inflammatory injury markers	Yes (macrophages)	Yes	Direct (virus-specific)	Direct antiviral effects demonstrated mainly in vitro; in vivo protection combines antiviral and immunomodulatory mechanisms
**Fibrosis-related** **pathways**	No	No	Yes	Extrapolated	No porcine fibrosis models directly testing curcumin currently available

**Table 5:** Integrative overview of curcumin-mediated modulation across stages of chronic liver disease and systemic metabolic axes. The down arrow (↓) indicates a decrease in level, while the up arrow (↑) indicates an increase in the level of a given process.

Disease Stage/Axis	Dominant Pathological Features	Key Modulated Processes by Curcumin	Mechanistic Interpretation (Integrative)	Porcine Relevance/Evidence Context
**NAFLD-like states**	Hepatic lipid accumulation; lipotoxic stress; subclinical inflammation	↓ Lipotoxic intermediates; ↑ metabolic stability	Curcumin stabilizes immunometabolic coupling by reducing inflammatory and oxidative pressure that secondarily drives dysregulated lipid flux	Porcine hepatic steatosis often secondary to inflammatory or endotoxin stress rather than caloric excess alone
**NASH-like** **pathology**	Inflammation-driven hepatocyte injury; immune-cell activation; cytokine amplification	↓ Inflammatory signal amplification; ↑ hepatocyte injury threshold	Curcumin modulates inflammatory gain, limiting transition from adaptive stress to irreversible hepatocyte damage without suppressing host immunity	Highly relevant in pigs where steatohepatitis commonly arises from infection- or endotoxin-driven inflammation
**Fibrosis**	Extracellular matrix deposition; stellate-cell activation; structural remodeling	↓ Pro-fibrotic microenvironment; ↓ remodeling persistence	Indirect disruption of inflammatory–fibrogenic coupling by attenuating upstream inflammatory and oxidative drivers	Fibrogenesis in pigs typically reflects unresolved inflammation rather than primary fibrotic disease
**Cirrhosis**	Architectural distortion; vascular remodeling; loss of metabolic reserve	↓ Secondary inflammatory and oxidative insults (supportive)	Adjunctive stabilization aimed at reducing episodic decompensation rather than reversing established structural damage	Limited porcine data; effects interpreted conservatively as supportive rather than disease-modifying
**Gut–liver axis**	Intestinal barrier dysfunction; microbial product translocation; portal inflammatory load	↑ Intestinal barrier integrity; ↓ endotoxin-driven hepatic inflammation	Reduction in portal inflammatory inputs lowers hepatic immune activation without requiring high systemic exposure	Highly relevant in pigs under intensive production conditions with frequent endotoxemia
**Gut–brain–liver axis**	Systemic inflammatory signaling; altered neuroendocrine regulation; stress responsiveness	↓ Systemic inflammatory tone; ↑ physiological homeostasis	Dampening of inflammatory spillover may normalize neuroendocrine–hepatic cross-talk affecting growth and stress responses	Direct porcine evidence limited, but mechanistically consistent with systemic inflammation models
**Lipid metabolism (cross-cutting)**	Dysregulated lipid uptake, processing, and export; lipid peroxidation	↓ Lipotoxic intermediates; ↑ metabolic resilience	Effects reflect stabilization of inflammatory and redox environments governing lipid-handling pathways	Porcine lipid dysregulation commonly linked to inflammation and oxidative stress rather than isolated metabolic overload
**Oxidative stress (cross-cutting)**	Elevated reactive species; mitochondrial stress; redox imbalance	↓ Oxidative pressure; ↑ cellular resilience	Reduction in oxidative burden raises hepatocyte tolerance to inflammatory and metabolic stress	Conserved across mammalian systems; indirect evidence supports relevance in pigs

**Table 6:** Evidence status of curcumin-based combination strategies in porcine and non-porcine liver models.

**Combination Strategy**	**Evidence Species**	**Model/Context**	**Evidence Status**
**Curcumin + mycotoxin binders**	Pig	Feed contamination/oxidative stress	Porcine-supported
**Curcumin + antioxidants (vit. E, selenium)**	Pig (limited)	Oxidative stress models	Indirect porcine
**Curcumin + UDCA**	Rodent	NASH	Extrapolated
**Curcumin + polyphenols (resveratrol, EGCG)**	Rodent/in vitro	Metabolic liver injury	Extrapolated

**Table 7:** Porcine studies investigating hepatoprotective mechanisms of curcumin. The down arrow (↓) indicates a decrease in level, while the up arrow (↑) indicates an increase in the level of a given process.

Ref.	Animal Model	Condition/Model	Curcumin Dose	Formulation/Route	Duration	Key Hepatic/ Infection-Relevant Outcomes	Mechanistic Pathways/ Targets
[79]	Growing pigs (IUGR)	IUGR-associated oxidative stress	200 mg/kg diet	Dietary curcumin	Day 26–115 of life (~89 days)	↓ lipid peroxidation; ↑ antioxidant enzymes	Nrf2 pathway; ↑ HO-1, GST, NQO1
[82]	Weaned piglets	LPS-induced liver injury & dyslipidemia	200 mg/kg diet	Dietary curcumin (98%)	28 days (LPS on days 18 & 28)	↓ AST, ↓ LDH; ↓ TC & TAG; ↓ liver weight	↓ SREBP-1c, ↓ SCD-1; apoptosis genes; m6A RNA methylation (METTL3/14)
[81]	PAMs + MARC-145 (porcine-derived in vitro)	PRRSV infection	5–15 μM	In vitro (DMSO vehicle)	1 h treatment; readouts up to 36 h p.i.	>90% ↓ viral replication	Inhibition of viral internalization/uncoating
[86]	Growing pigs	Safety/subchronic exposure	60–1551 mg/kg BW/day *(Turmeric oleoresin)*	Dietary oleoresin	102–109 days	Dose-related ↑ liver weight	Toxicological safety (not hepatoprotection)

**Table 8:** Clinical applications of *Curcuma longa* and curcumin. The down arrow (↓) indicates a decrease in level, while the up arrow (↑) indicates an increase in the level of a given process.

Compound	Clinical Indication	Target Condition	Proposed Mechanism	Type of Evidence	Key Outcomes	Limitations	Ref.
***Curcuma longa* (turmeric tonic)**	Anti-inflammatory, symptom relief	Scalp psoriasis (*Psoriasis vulgaris*)	Anti-inflammatory and immune modulation effects reducing erythema, scaling, induration	Randomized, placebo-controlled clinical trial (RCT)	↓ PASI score; improved dermatology life quality (DLQI) compared to placebo	Topical formulation; study size, formulation specificity	[122]
**Curcumin**	Anti-inflammatory, immunomodulatory therapy	Oral lichen planus	Inhibition of inflammatory mediators; antioxidant and immune-modulating effects	Clinical trial (human study)	Reduction in lesion severity and pain; clinical improvement with good tolerability	Limited sample size; short follow-up	[125]
**Curcumin**	Anti-inflammatory therapy	Chronic inflammatory conditions (human subjects)	Anti-inflammatory and antioxidant activity; modulation of inflammatory mediators	Clinical study (human intervention)	Improvement of clinical symptoms; good tolerability	Lack of large randomized controlled trials; limited sample size	[126]
**Curcumin**	Anti-inflammatory, immunomodulatory therapy	Oral lichen planus	Anti-inflammatory and antioxidant effects; modulation of immune response	Clinical study (human intervention)	Reduction in lesion severity and oral pain; good tolerability	Limited sample size; lack of placebo-controlled randomization	[127]
**Curcumin**	Anti-inflammatory, immunomodulatory therapy	Oral lichen planus	Anti-inflammatory and antioxidant activity; modulation of immune response	Randomized clinical trial (human study)	Comparable clinical efficacy to corticosteroids; reduction in lesion severity and pain	Limited sample size; short follow-up	[127]
***Curcuma longa* (hydroalcoholic extract; oral)**	Cardiometabolic risk modulation (atherogenesis prevention—markers)	Atherogenic lipid profile (apoB/apoA ratio) in healthy subjects	Antioxidant/anti-inflammatory effects; modulation of lipid/apolipoprotein profile	Human intervention study (30-day supplementation)	↓ LDL & apoB; ↑ HDL & apoA; ↓ apoB/apoA ratio; no notable GI side effects reported	Surrogate biochemical endpoints; no clinical cardiovascular outcomes; non-RCT design	[130]

**Table 9:** Traditional applications of *Curcuma longa* and knowledge gaps. The down arrow (↓) indicates a decrease in level, while the up arrow (↑) indicates an increase in the level of a given process.

Traditional Application of *C. longa*	Reported Scientific Evidence	Identified Knowledge Gaps	Key References
**Anti-bacterial**	Attenuation of LPS-driven inflammatory consequences rather than direct bactericidal action; modulation of gut microbiota composition with reduction in pathogenic taxa and enrichment of short-chain fatty acid–producing bacteria; regulation of gut barrier–immune axis via intestinal alkaline phosphatase and cytokine signaling	Lack of direct in vivo bactericidal evidence; effects largely indirect and microbiota-mediated; species-, formulation-, and time-dependent outcomes; limited clinical validation	[52,57,77,86]
**Antiviral**	Inhibition of viral replication in vitro; interference with lipid metabolism required for late stages of viral replication (CSFV); impairment of viral fusion and uncoating in host cells (PRRSV); reduction in viral load in porcine macrophages and cell lines; suppression of viral transcription via epigenetic and transcription factor–dependent mechanisms (HBV, HPV)	Evidence largely limited to in vitro and preclinical models; lack of in vivo efficacy data in pigs for most viruses; virus- and stage-specific mechanisms; absence of standardized dosing and clinical validation; limited data on systemic antiviral efficacy	[56,83,85]
**Antiparasitic**	Direct antiparasitic activity in vitro; induction of oxidative stress in adult liver flukes (*Fasciola gigantica*); reduction in parasite antioxidant defenses (glutathione, SOD, GST); tegumental and protein damage leading to reduced viability; concurrent hepatoprotective effects via activation of Nrf2-mediated detoxification and antioxidant pathways	Evidence limited primarily to in vitro parasite models; lack of in vivo efficacy and dosing data; unclear selectivity between parasite toxicity and host safety; limited translational relevance to field infections	[58,59]
**Antioxidant**	Direct scavenging of ROS and RNS; reduction in lipid peroxidation markers (e.g., MDA); activation of Nrf2/ARE signaling with induction of cytoprotective enzymes (HO-1, NQO1, GST); suppression of upstream ROS generation (NADPH oxidase inhibition); reinforcement of endogenous antioxidant capacity in inflammatory and oxidative injury models, including porcine in vivo hepatic stress	Antioxidant effects often inferred from biomarker shifts rather than functional recovery; limited correlation with histopathology or liver function endpoints in some models; bioavailability constraints; need for standardized dosing and outcome measures	[38,39,40,41,42,50,53,54]
**Anti-fungal**	In vitro inhibition of growth of pathogenic fungi (mainly *Candida* spp. and filamentous fungi); disruption of fungal cell membrane integrity and ergosterol biosynthesis; induction of oxidative stress and mitochondrial dysfunction in fungal cells; inhibition of adhesion and biofilm formation; enhanced antifungal efficacy observed with nanoparticle-based formulations	Evidence restricted to in vitro models; absence of in vivo or clinical antifungal efficacy data; high variability depending on fungal species, formulation, and dose; unclear selectivity and host safety; limited relevance to systemic infections	[37,38,39,40]
**Anti-cancer**	Cytotoxic activity of ethanolic rhizome extracts and isolated curcuminoids against multiple cancer cell lines with low toxicity toward non-malignant cells; inhibition of proliferative and survival signaling pathways (PI3K/Akt/mTOR, MAPK); induction of mitochondrial apoptosis (Bax/Bcl-2 shift, cytochrome c release, caspase activation); suppression of angiogenic and inflammation-driven tumor-supportive programs; modulation of drug efflux transporters (P-gp/ABCB1, BCRP/ABCG2) potentially increasing intracellular drug exposure	Evidence largely restricted to in vitro and preclinical models; lack of robust in vivo and clinical efficacy data; strong dependence on formulation and curcuminoid composition; poor oral bioavailability; unclear therapeutic window and relevance to hepatic malignancies in vivo	[38,92,93,94]
**Anti-inflammatory**	Inhibition of NF-κB–centered inflammatory signaling; suppression of pro-inflammatory cytokine transcription (TNF-α, IL-1β, IL-6); modulation of TLR4–NF-κB coupling; regulation of AP-1 and MAPK pathways; inhibition of JAK/STAT signaling via induction of SOCS1/SOCS3; promotion of anti-inflammatory cytokine profiles (IL-10, IL-4); reduced expression of inducible inflammatory enzymes (iNOS, COX-2, 5-LOX) and downstream eicosanoid production	Direct validation of NF-κB modulation in porcine liver tissue remains limited; mechanistic evidence largely derived from non-hepatic or non-porcine models; dose- and context-dependent effects; limited linkage between pathway modulation and functional liver outcomes in vivo	[38,43,44,48,49,50,52]
**Antifibrinogenic**	In vivo attenuation of coagulation dysregulation in endotoxemia; reduced plasma fibrinogen consumption and prevention of thrombocytopenia; marked reduction in fibrin deposition in renal glomeruli; decreased TNF-α–driven coagulation activation in LPS-induced disseminated intravascular coagulation (DIC) rat model	Evidence limited to acute endotoxemia models in rodents; lack of data in chronic fibrotic or liver-specific fibrin deposition contexts; unclear contribution of direct fibrinolytic effects versus secondary anti-inflammatory mechanisms; absence of porcine and clinical validation	[41]
**Radioprotective**	Reduced radiation-induced damage; activation of antioxidant defense pathways in vivo; improved survival and attenuation of oxidative stress and inflammation in irradiated animal models	Evidence limited to preclinical models; dose- and formulation-dependent effects; poor bioavailability of native curcumin; lack of clinical validation	[112,113]
**Immunomodulating**	Modulation of innate and adaptive immune responses; attenuation of TLR4-driven inflammatory signaling; inhibition of JAK/STAT pathways via induction of SOCS1/SOCS3; polarization of macrophages and microglia toward anti-inflammatory phenotypes; suppression of dendritic cell maturation and antigen presentation; rebalancing of cytokine profiles (↓ TNF-α, IL-1β, IL-6; ↑ IL-10, IL-4); indirect immune modulation via gut barrier reinforcement and microbiota remodeling	Predominantly preclinical evidence from non-porcine and non-hepatic models; limited direct validation in porcine immune cells and liver-resident macrophages (Kupffer cells); context- and dose-dependent effects; unclear contribution of microbiota-mediated versus cell-intrinsic mechanisms; lack of clinical immunomodulatory endpoints	[43,48,49,50,52,57,77,86]

**Table 10:** Recommended biomarker and endpoint panel for porcine liver dysfunction studies.

Domain	Core Endpoints (Minimum)	Mechanistic/Confirmatory Endpoints (Recommended)	Notes for Interpretation
Hepatocellular injury	ALT, AST	LDH (optional), hepatocyte death assays where relevant	Enzymes alone do not distinguish reversible leakage vs. necrosis; interpret with histology.
Cholestasis/excretory function	ALP, GGT, total bilirubin	Total bile acids (high value), bile acid profiling (advanced)	Particularly relevant in cholestatic/toxin models and for bile-flow–linked injury.
Oxidative stress	MDA/TBARS (minimum)	GSH, GSSG, GSH/GSSG ratio; SOD, CAT, GPx; antioxidant response targets (e.g., HO-1/NQO1/GST)	Use at least one damage marker plus one antioxidant capacity measure.
Inflammation	TNF-α, IL-6, IL-1β	NF-κB pathway readouts; inflammasome-axis indicators (NLRP3-related); macrophage/Kupffer-cell activation/infiltration proxies	Cytokines without pathway/cellular context are prone to over-interpretation.
Histology (mandatory)	H&E with standardized scoring	Steatosis grading; necrosis scoring; apoptosis assays when claimed; fibrosis staining (collagen) for chronic models; α-SMA when stellate activation is claimed	Blinded scoring and clear sampling strategy are critical; zonal injury can bias results.
Model-driven add-ons	Growth/performance where relevant	Pathogen burden proxies in infection models; collagen content/ECM markers in fibrosis models	Add only what matches the dominant mechanism of the model.

## Data Availability

No new data were created or analyzed in this study. Data sharing is not applicable to this article.
